# Implications of Laboratory Tests in Disease Grading and Death Risk Stratification of COVID-19: A Retrospective Study in Wuhan, China

**DOI:** 10.3389/fmed.2021.629296

**Published:** 2021-02-19

**Authors:** Yang Bai, Enxin Wang, Shoujie Zhao, Jing Li, Yejing Zhu, Yongchao Zhang, Liang Cao, Haitao Liu, Yushu Dong, Fang Wang, Guobiao Liang, Lei Liu

**Affiliations:** ^1^Department of Gastroenterology, Tangdu Hospital of Fourth Military Medical University, Xi'an, China; ^2^Department of Neurosurgery, General Hospital of Northern Theater Command, Shenyang, China; ^3^Department of Medical Affairs, Air Force Hospital of Western Theater Command, Chengdu, China; ^4^Department of General Surgery, Tangdu Hospital of Fourth Military Medical University, Xi'an, China; ^5^Department of Digestive Disease, Shanxi Bethuen Hospital of Shanxi Academy of Medical Sciences, Taiyuan, China; ^6^Department of Traditional Chinese Medicine, Tangdu Hospital of Fourth Military Medical University, Xi'an, China; ^7^Department of Cardiology, Xijing Hospital of Fourth Military Medical University, Xi'an, China; ^8^Department of Dermatology, The First Affiliated Hospital of Sun Yat-sen University, Guangzhou, China; ^9^Division of Dermatology, Department of Medicine, Washington University School of Medicine, St. Louis, MO, United States

**Keywords:** SARS-CoV-2, clinical classification, risk-stratification, prediction model, IL-6

## Abstract

**Background:** Although laboratory tests have become an indispensable part in clinical practice, its application in severity classification and death risk stratification of COVID-19 remains unvalidated. This study aims to explore the significance of laboratory tests in the management of COVID-19.

**Methods:** In 3,342 hospitalized patients with COVID-19, those of mild or moderate subtype were categorized into the non-severe group, while those of severe or critical subtype were categorized into the severe group. Initial laboratory data were analyzed and compared according to disease severity and outcome. Diagnostic models for the severe group were generated on risk factors identified by logistic regression and receiver operating characteristic (ROC) analyses. Cox regression and ROC analyses on risk factors were utilized to construct prognostic models.

**Results:** In identification of patients in the severe group, while age, neutrophil-to-lymphocyte ratio, and α-hydroxybutyrate dehydrogenase were identified as independent predictors, the value of combination of them appears modest [area under the curve (AUC) = 0.694]. Further ROC analyses indicated that among patients in the severe group, laboratory indices had a favorable value in identifying patients of critical subtype rather than severe subtype. For death outcome, IL-6, co-existing cerebrovascular disease, prothrombin time activity, and urea nitrogen were independent risk factors. An IL-6 single-parameter model was finalized for distinguishing between fatal and recovered individuals (AUC = 0.953). Finally, a modified death risk stratification strategy based on clinical severity and IL-6 levels enables more identification of non-survivors in patients with non-critical disease.

**Conclusions:** Laboratory screening provides a useful tool for COVID-19 management in identifying patients with critical condition and stratifying risk levels of death.

## Introduction

Coronavirus disease 2019 (COVID-19) is a pandemic infectious disease caused by severe acute respiratory syndrome-coronavirus-2 (SARS-CoV-2) (1). Since its outbreak from Wuhan in December 2019, COVID-19 has affected over 35 million patients and caused more than 1 million deaths according to the latest report from the World Health Organization (2). The clinical spectrum of COVID-19 varies from asymptomatic or paucisymptomatic forms to severe clinical conditions characterized by dyspnea and lethal complications such as acute respiratory distress syndrome (ARDS), multi-organ failure, and septic shock (3, 4). While mild or moderate disease was exhibited by ~80% patients, severe and critical conditions were diagnosed in the remaining 20% (5). Although the accurate case-fatality rate (CFR) across various disease severity remains unclear, the CFR in patients with critical disease was reported up to 49% (3, 5). Therefore, precise identification of disease severity and underlying risk factors for mortality is of paramount importance to initiate individualized therapeutics and improve patient outcomes.

Laboratory tests performed on blood samples reflect individual physiological and biochemical states. Accumulating laboratory data have revealed a variety of abnormities such as coagulopathy, myocardial injury, liver damage, kidney injury, and immune dysfunction in patients with severe COVID-19 (6–8), particularly in those fatal cases (9–11). Despite the significance in COVID-19, laboratory items have not been included in the current clinical classification of COVID-19, which is mainly based on clinical manifestations and radiologic features (12). Given that disease severity is directly linked to treatment decision and prognosis, we hypothesized that, in addition to current clinical criteria, abnormal laboratory variables may provide an alternative tool to grade patients and, meanwhile, predict survival. Surprisingly, few studies have reported this before. The first area that experienced COVID-19 outbreak, Wuhan, has a large number of patients on whom broad and basic laboratory screening was exclusively performed. Therefore, we revisited patient datasets in Wuhan to investigate the significance of laboratory tests in disease grading along with the prognosis of COVID-19.

## Methods

### Study Participants

We reviewed a total of 3,477 medical records of COVID-19 from Wuhan Huoshenshan Hospital, Hubei Maternal and Child Health Hospital, and General Hospital of Central Theater Command from 5 February to 15 March 2020. These three tertiary hospitals, in Wuhan of Hubei Province, were specifically requisitioned to treat patients with COVID-19 during the outbreak in China.

**Figure 1:**
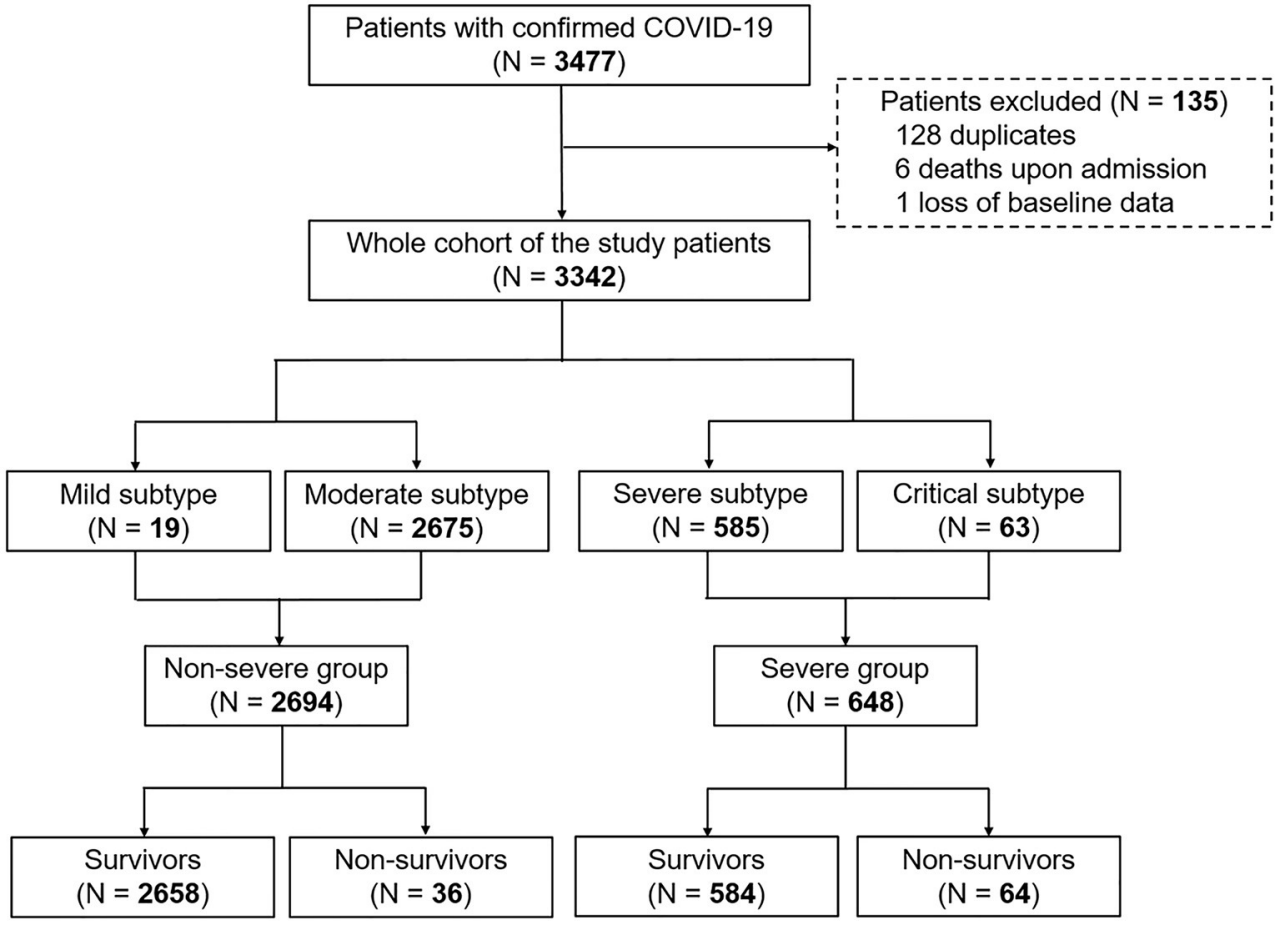
Flowchart of patient recruitment.

The diagnosis of COVID-19 was based on World Health Organization interim guidance (13). Disease severity was defined according to the guideline of diagnosis and management for COVID-19 (sixth edition, in Chinese) released by the National Health Commission of China. The mild subtype was diagnosed if patients had slight clinical symptoms without pneumonia on radiography. The moderate subtype was confirmed when patients presented with fever and/or respiratory symptoms plus pneumonia on radiography. While patients were classified into the severe subtype if they exhibited dyspnea (respiratory frequency ≥ 30/min), blood oxygen saturation ≤ 93%, or PaO_2_/FiO_2_ ≤ 300 mmHg, patients with respiratory failure, multi-organ failure or shock and requirement of mechanical ventilation and intensive care unit admission were categorized into the critical subtype (12). In this study, patients with mild or moderate disease were classified into the non-severe group, whereas the severe group included those with severe or critical condition (Figure 1). All patients were followed up till recovery or death.

### Data Collection

Clinical data including demography, medical history, clinical manifestations, laboratory blood test results, and outcomes were collected and independently reviewed by two attending physicians. We focused on the comprehensive laboratory results including the following seven categories: complete blood cell count, coagulative state, myocardial injury markers, liver function markers, kidney function markers, electrolyte and glucose test, as well as inflammatory factors including C-reactive protein (CRP) and IL-6 from each patient on admission (Supplementary Table 1).

### Statistical Analysis

No imputation was made for variables with missing data. Quantitative data with non-normal distribution were expressed in median [interquartile range (IQR)] and statistically compared by Mann-Whitney U non-parametric test. Percentage (%) of enumeration data were calculated and compared using the χ^2^ test or Fisher's exact test. Survival curves were plotted using the Kaplan–Meier method with log-rank test.

Risk factors associated with disease severity in demography and laboratory variables were analyzed using univariate logistic regression analysis followed by receiver operating characteristic (ROC) curve analyses. To avoid excessive laboratory variables in subsequent multivariate analyses, one or two risk factors in each category of laboratory tests meeting the following requirements were selected: (1) significant variables identified in univariate analyses; (2) variables with scientific and clinical merits or proven to relate to disease severity in prior studies; and (3) variables with high AUC value identified in ROC analyses. Considering that the elderly, especially those with comorbidities, could easily progress from dyspnea to critical condition and even death (5, 14), all significant demographic variables identified in univariate analyses were selected as potential confounding variables in the multivariable models with a forward stepwise approach. Similarly, survival prediction models were developed using univariate and multivariable Cox regression analyses.

Further, a nomogram for predicting survival was built and evaluated by the AUC value and calibration plots. All statistical analyses were performed using SPSS software (version 22.0, IBM Corp) and R software (version 3.3.1, R Foundation). *P* < 0.05 were considered statistically significant.

## Results

### Patient Characteristics, Laboratory Findings, and Clinical Outcomes

Data of 3,342 patients with COVID-19 from a total of 3,477 medical records were analyzed (Figure 1). Overall, 19 patients, 2,675 patients, 585 patients, and 63 patients were classified into mild subtype, moderate subtype, severe subtype, and critical subtype, respectively. Accordingly, 2,694 patients (80.6%) and 648 patients (19.4%) were categorized into the non-severe group and severe group, respectively (Figure 1). The death rate was 2.99% (100/3,342) in the entire cohort. The CFR of 9.88% (64/648) in the severe group was significantly higher than that of 1.34% in the non-severe group (*P* < 0.001; Figure 1).

Compared with the non-severe group, the severe group had an older median age and was composed of a higher proportion of male and those with various comorbidities (Supplementary Table 1). By analyzing laboratory blood tests, we found that coagulopathy, myocardial injury, kidney injury, and increased CRP and IL-6 levels were exhibited more frequently in the severe group than in the non-severe group (Supplementary Figure 1A). After separating patients according to outcome, we observed a similar tendency of the above demographic and laboratory characteristics in non-survivors compared to survivors. Notably, liver injury with a higher frequency in non-survivors was the only feature that was not significantly different between the severe group and the non-severe group (Supplementary Figure 1B; Supplementary Table 1).

**Figure 2:**
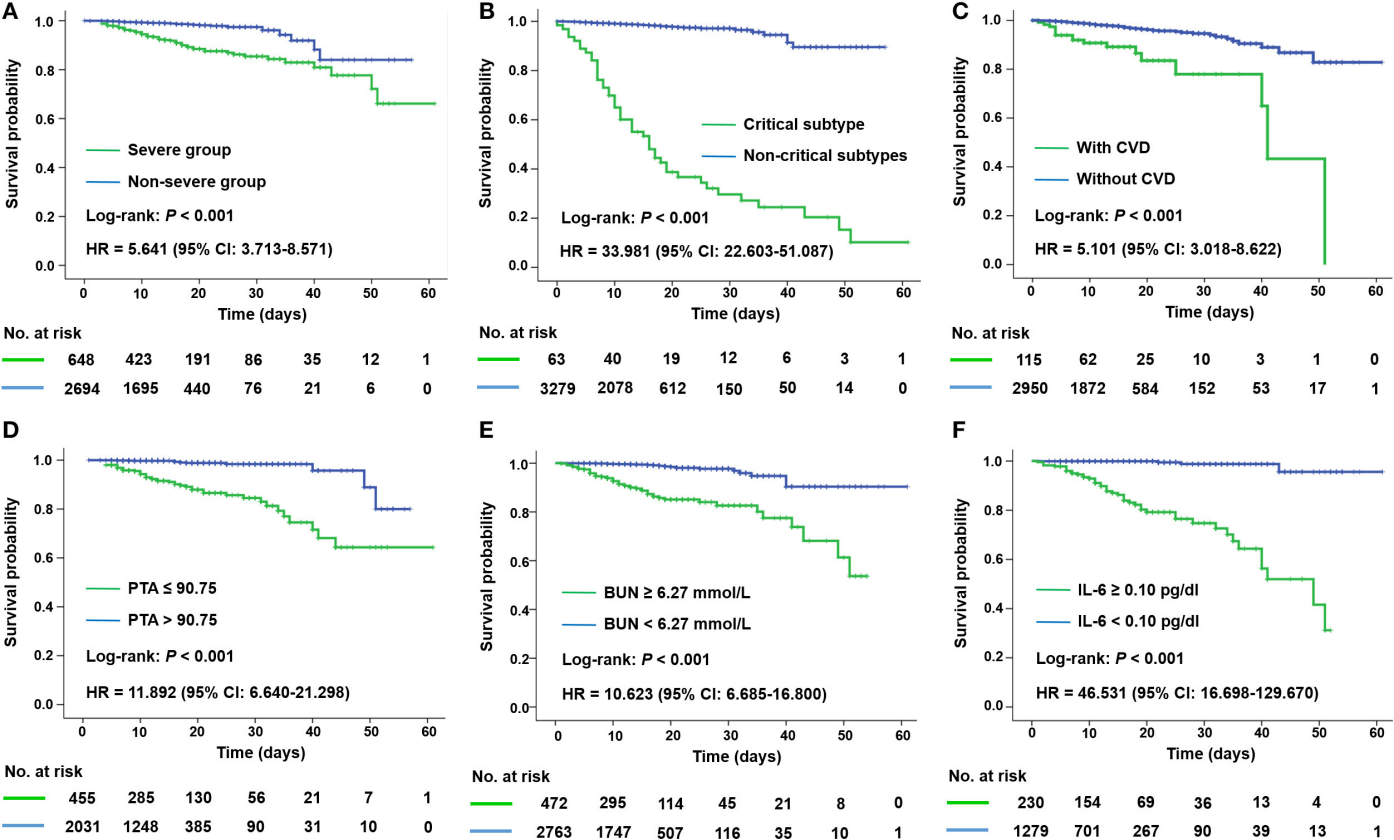
Kaplan–Meier plots for disease severity and different prognostic factors of all patients with COVID-19. Mortality was significantly higher in patients of the severe group **(A)**, of critical subtype **(B)**, and with CVD **(C)**, PTA ≤ 90.75 **(D)**, BUN ≥ 6.27 mmol/L **(E)**, and IL-6 ≥ 0.10 pg/dl **(F)** according to corresponding ROC cutoffs. BUN, blood urea nitrogen; CVD, cerebrovascular disease; HR, hazard ratio; IL-6, interleukin-6; PTA, prothrombin activity.

As expected, patients in the severe group had a longer hospitalization stay [median (IQR), 15 (8–23) vs. 13 (8–18) days; *P* < 0.001] than those in the non-severe group. A significantly escalated risk of mortality was also revealed by Kaplan–Meier curve for patients in the severe group [hazard ratio (HR), 5.641; Figure 2A] or of critical subtype (HR, 33.981; Figure 2B).

### Predictive Laboratory Factors for Identifying Patients in Severe Group

Initial univariate logistic analysis identified 46 significant risk factors for the severe group (Supplementary Table 2). Among them, the onset age had the highest predictive accuracy, but with the AUC value of only 0.657 (Supplementary Table 2). Thereafter, a multivariate model, including all significant demographic variables and 10 laboratory variables with highest AUCs that represent multi-organ injury, was established, indicating that age [odds ratio (OR), 1.032], neutrophil-to-lymphocyte ratio (NLR; OR, 1.090), and α-hydroxybutyrate dehydrogenase (OR, 1.004) were independent risk factors for severe COVID-19 (Supplementary Table 3).

**Figure 3:**
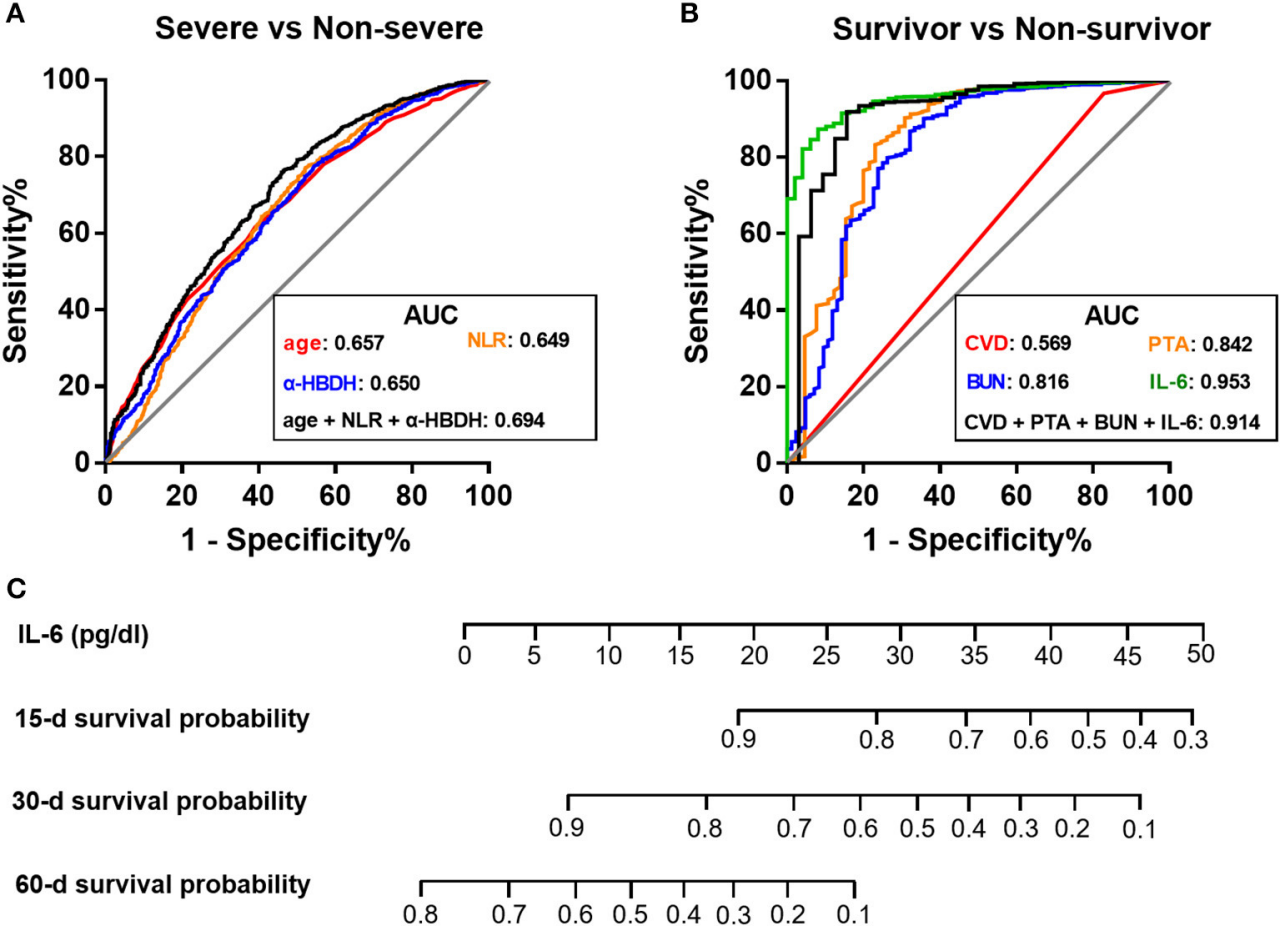
Comparison of receiver operating characteristic curves of laboratory models for disease grading and prognosis of COVID-19. **(A,B)** ROC curves for the classification of severe and non-severe medical conditions **(A)**, and the prediction of 60-day survival probability **(B)**. **(C)** Prognostic nomogram based on IL-6 for predicting survival probability. AUC, area under the curve; BUN, blood urea nitrogen; CVD, cerebrovascular disease; α-HBDH, α-hydroxybutyrate dehydrogenase; IL-6, interleukin-6; NLR, neutrophil-to-lymphocyte ratio; PTA, prothrombin activity; ROC, receiver operating characteristic.

Further, we developed three single-parameter models and one multi-parameter model based on the above independent predictors to differentiate the severe group and the non-severe group (Supplementary Table 4). However, these models possessed undesirable discrimination as the highest AUC in Model 4 was only 0.694 (Figure 3A), suggesting that laboratory data may not be strongly associated with clinical severity classification.

### Prognostic Laboratory Factors for Mortality in the Whole Cohort

Univariate Cox regression analysis identified 48 significant risk factors for mortality in all patients (Supplementary Table 5). We incorporated 21 items with relatively high AUC into a multivariable model and found that cerebrovascular disease (CVD; HR, 6.162), prothrombin activity (PTA; HR, 0.912), blood urea nitrogen (BUN; HR, 1.207), and IL-6 (HR, 1.085) were independent predictors for fatality (Supplementary Table 6). Kaplan–Meier analysis (Figures 2C–F) revealed a poorer prognosis in patients with pre-existing CVD, decreased PTA (≤90.75 vs. >90.75), elevated BUN (≥6.27 vs. <6.27 mmol/L), or elevated IL-6 (≥0.10 vs. <0.10 pg/dl) according to corresponding ROC cutoffs.

Further, four single-parameter models and four multi-parameter models were developed (Supplementary Table 7). Unlike the models in differentiation of disease severity, models aiming at predicting survival possessed a favorable performance (Figure 3B). Notably, among these candidates, a single-parameter model based on IL-6 levels had the highest discrimination (AUC = 0.953) and a good calibration (Figure 3B; Supplementary Figure 2). At the optimal cutoff of 0.10 pg/dl, the sensitivity and specificity was 91.8% and 86.3%, respectively. Therefore, a nomogram based on serum IL-6 levels was constructed to predict survival for further clinical practice (Figure 3C).

### Prognostic Value of IL-6 for Mortality in Severe Group

Given that severe patients have higher risk of poor prognosis (3, 5), we sought to investigate whether IL-6 remains effective in survival prediction specifically among patients in the severe group. Again, non-survivors exhibited significantly higher levels of IL-6 than those survivors (*P* < 0.001; Supplementary Table 8). Subsequent Cox regression analyses revealed that IL-6 (HR, 1.114), together with pre-existing chronic kidney disease (CKD), increased NLR, and decreased PTA, was the independent predictor for fatal outcome in the severe group (Supplementary Tables 9, 10; Supplementary Figure 3). Among all the candidate models (Supplementary Table 11), IL-6 still had a relatively high performance (AUC = 0.914), with a sensitivity of 87.5% and a specificity of 84.7% at the cutoff of 0.17 pg/dl.

### Predictive Value of IL-6 for Patients of Varying Severity

**Figure 4:**
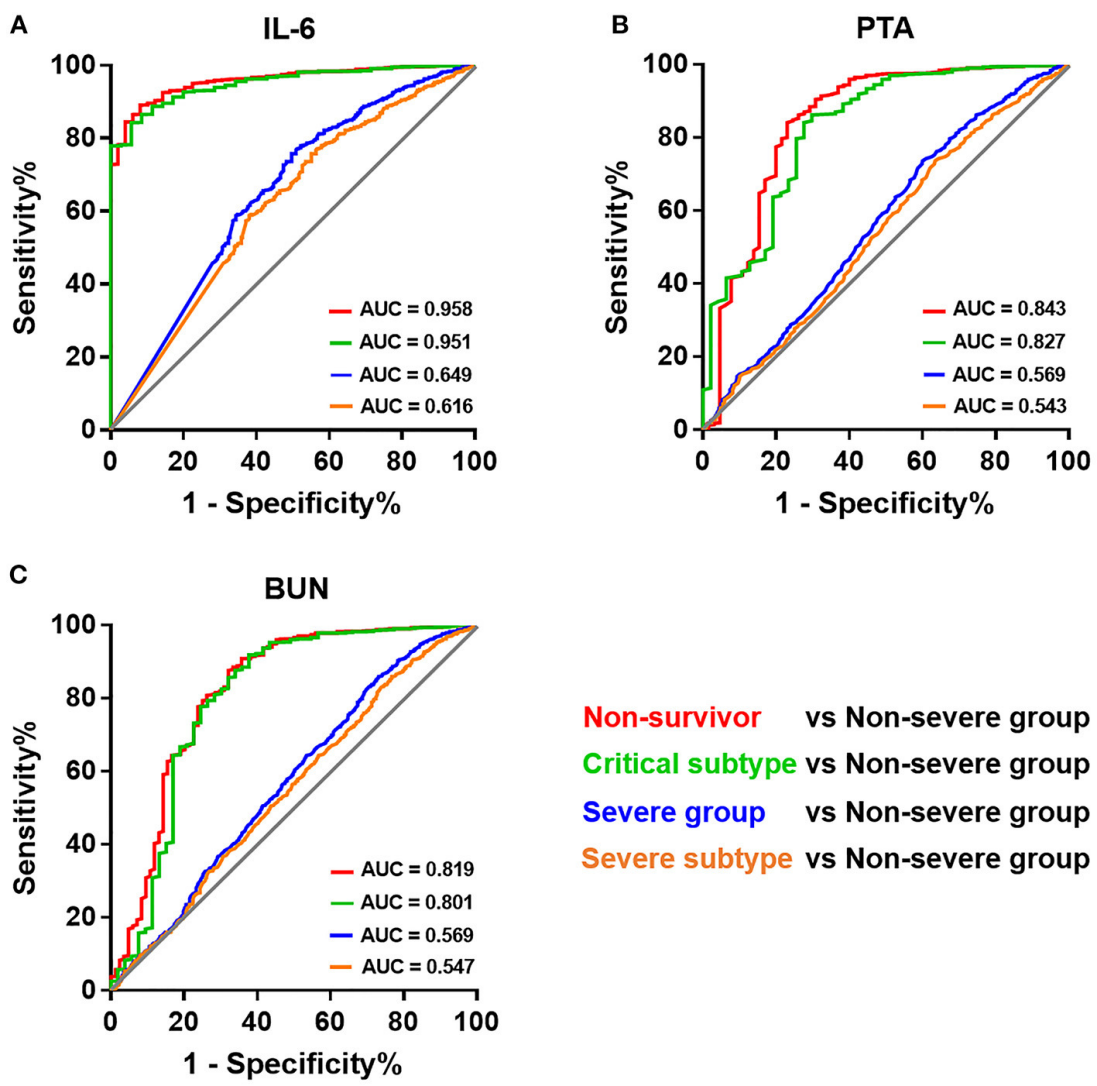
Receiver operating characteristic curves of different laboratory prognostic factors for identifying patients of varying severity. Receiver operating characteristic curves of IL-6 **(A)**, PTA **(B)**, and BUN **(C)** for identifying patients of varying severity. BUN, blood urea nitrogen; IL-6, interleukin-6; PTA, prothrombin activity.

We next sought to investigate the reason for the discrepancy in predictive value of laboratory indices when distinguishing non-survivors and those in the severe group. IL-6, PTA, and BUN, independent laboratory predictors for overall mortality mentioned before (Supplementary Table 6), were adopted for the analyses herein. The predictive values of IL-6 for differentiating four subpopulations of COVID-19 patients, including non-survivors, critical subtype, severe group (including both critical and severe subtypes), and severe subtype, from the non-severe group were compared. Strikingly, we observed a substantial decline in AUC from non-survivors (AUC = 0.958) and critical subtype (AUC = 0.951) to the severe group (AUC = 0.649) and severe subtype (AUC = 0.616; Figure 4A). Similar phenomenon was also present in PTA and BUN (Figures 4B,C). In addition, IL-6 outperformed PTA and BUN when identifying those with fatal outcome or in critical condition (Figure 4). Taken together, these results indicate that laboratory results have a favorable value in identifying patients of critical subtype rather than severe subtype. Since patients of severe subtype occupied the vast majority of the severe group (585/648), its role in disease grading was weakened by the inefficacy in identifying severe subtype.

### A Modified Risk Stratification Strategy for COVID-19

**Figure 5:**
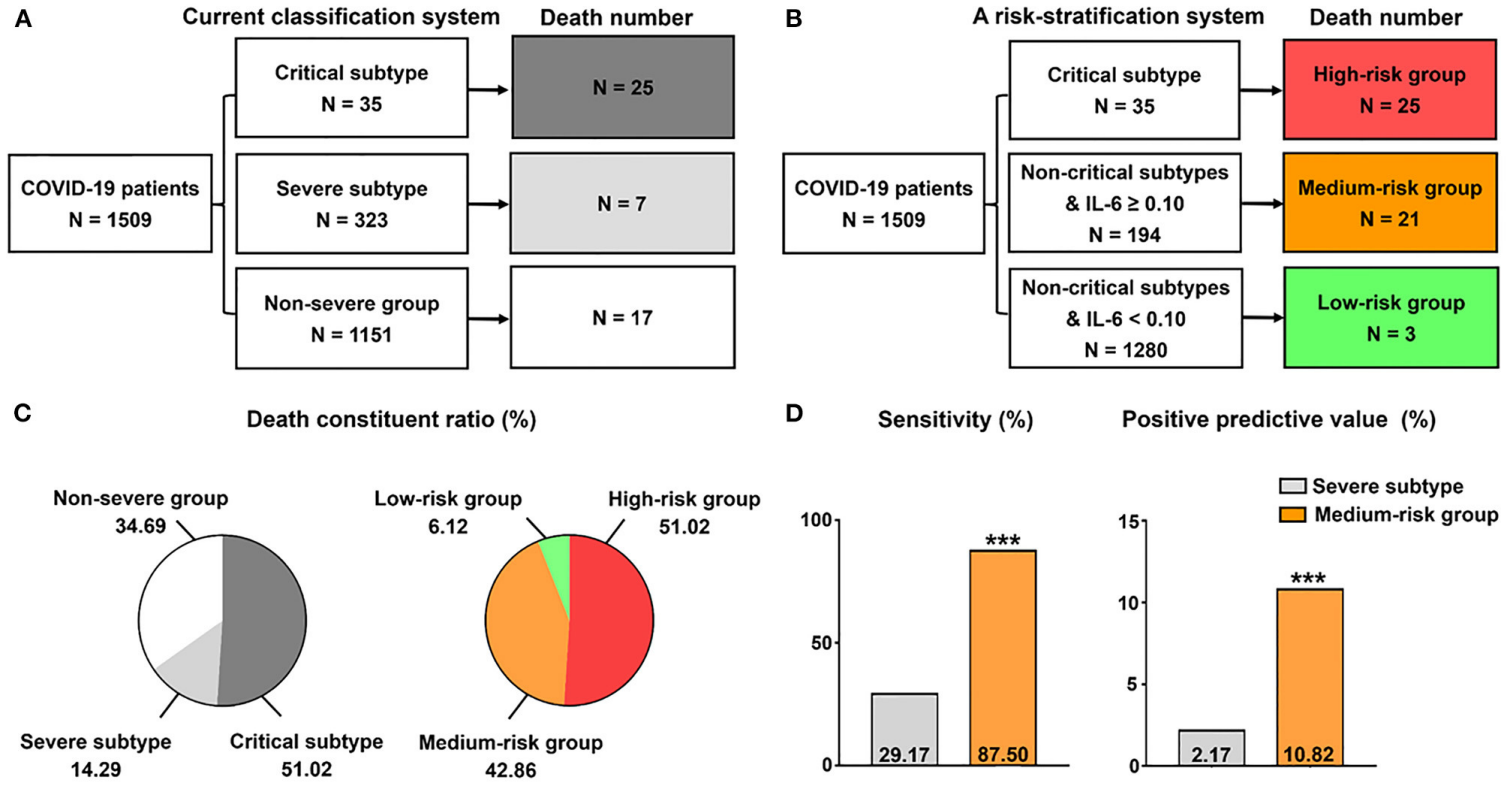
A modified grading strategy for COVID-19. **(A)** Current classification system. **(B)** A risk stratification system on the basis of IL-6 and current classification system. **(C)** Death constituent ratio of the two grading methods. The data in the pie chart indicate death constituent ratio (%) in each group. **(D)** Comparison of the efficacy of the medium-risk group in identifying non-critical patients with fatal outcome between the two methods. ***P < 0.001.

Based on the current clinical classification system, we found that CFR was not dramatically different between patients of severe subtype (3.08%) and in the non-severe group (1.34%). Thus, we sought to investigate whether death risk stratification could be improved with the introduction of laboratory variables. Given the good performance in identifying patients with fatal outcome and in critical condition, we integrated IL-6 assessment into the current clinical classification system. In a cohort of 1,509 patients with the initial IL-6 test, the non-severe group, severe subtype, and critical subtype had 1,151 (CFR = 1.48%, 17/1,151), 323 patients (CFR = 2.17%, 7/323), and 35 patients (CFR = 71.43%, 25/35; Figure 5A), respectively. Given the dramatically high CFR in patients of critical subtype, we ranked high-risk level to them without further modifications. Therefore, the death cases in the high-risk group occupied 51.02% (25/49) of total death cases (Figures 5B,C). In addition, 30 patients had IL-6 levels ≥ 0.1 pg/dl in this group. Further, using the cutoff value of 0.1 pg/dl, we found 1,280 patients with IL-6 levels < 0.1 pg/dl (CFR = 0.23%, 3/1,280) and 194 patients with IL-6 levels ≥ 0.1 pg/dl (CFR = 10.82%, 21/194) in those non-critical patients (Figure 5B). By introducing IL-6 levels, we surprisingly found that the death composition ratio was altered, in which patients with IL-6 levels ≥ 0.1 pg/dl took 42.86%, while those with IL-6 levels < 0.1 pg/dl occupied just 6.12% (Figure 5C). Therefore, low-risk and medium-risk groups were defined using the IL-6 of 0.1 pg/dl (Figure 5B). Compared with clinical classification, this strategy could identify more non-critical patients with fatal outcome in the medium-risk group, with a higher sensitivity (87.50 vs. 29.17%; *P* < 0.001) and positive predictive value (10.82 vs. 2.17%; *P* < 0.001; Figure 5D).

## Discussion

Our study determines the associations of basic laboratory screening with disease severity and prognosis of COVID-19. One surprising finding is that laboratory variables, alone or in combination, had a better performance in predicting survival than identifying patients in the severe group. Further analysis indicated that laboratory tests showed excellent performance in identifying patients of critical subtype rather than severe subtype. One possible explanation is that patients of severe subtype displayed only symptoms of hypoxia, instead of ARDS, multi-organ failure, or septic shock that frequently occurred in critical or deceased patients (10, 11), so no dramatic change was induced in these indices reflecting inflammation, multi-organ function, and homeostasis. Therefore, the inclusion of laboratory test should be considered for the diagnosis of critical COVID-19.

In accordance with prior studies concerning prognostic models of COVID-19 (15), we found that laboratory tests showed strong advantages in predicting survival, among which IL-6 stands out as the most appealing one owing to its superior discrimination. The presence of raised circulating levels of IL-6 has been shown closely relating to disease deterioration and fatal outcome of COVID-19 (8, 16–19). Our results support previous findings by finalizing a single-parameter IL-6 prognostic nomogram. Although it did not possess the highest discrimination compared with previous models (summarized in Supplementary Table 12), it may outperform them for its simplicity in clinical practice.

The classification of COVID-19 guides management decisions (12), but may not closely relate to risk stratification, owing to no huge difference observed in CFR between the severe subtype and the non-severe group. Therefore, we established a grading system for COVID-19 by combining serum IL-6 levels and current classification criteria. Excitingly, this modification was capable of identifying more non-critical patients with fatal outcome in the medium-risk group. Since our study focuses on routine bloodwork tests, whether the collaboration of IL-6 and other specific laboratory tests (20), such as virus tilter measurements, anti-SARS-CoV-2 antibody levels, and other immunological biomarkers could optimize the risk stratification of COVID-19 is an interesting question of future inquiries.

Severe COVID-19 is considered as a virally induced hyper-inflammatory condition with multi-organ involvement *via* the cytokine storm (20). IL-6 has been recognized as an important pro-inflammatory cytokine involved in this process, which impairs immune cell cytotoxicity, maintains antigen stimulation, and leads to sustained cytokine production (21, 22). The finalized IL-6 nomogram highlights cytokine storm as a core mechanism for COVID-19-related death, which is further supported by the fact that nomograms incorporating cytokine indices dominated the top of the ranking in predictive value for mortality (Supplementary Table 12). It is worth mentioning that IL-6 does not contradict with other laboratory indicators reflecting multi-organ function in prior prognostic models (Supplementary Table 12), since cytokine storm is the main culprit for multi-organ injury in COVID-19 (20). Hence, the control of cytokine storm is specifically emphasized in the treatment of critical patients (23).

The etiology of kidney injury is multifactorial, including direct cytopathic effect of SARS-CoV-2, cytokine storm, and systemic effects of lung inflammation (24, 25). Abnormal kidney function upon admission is considered a negative prognostic factor for survival (26, 27). Patients with CKD were also reported to have a poorer prognosis (28), since they were in a pro-inflammatory state with deficits in immune function and thus vulnerable to respiratory infection (29). Herein, we identified CKD and BUN as independent predictors for mortality, supporting the pivotal position of kidney damage in pathophysiology of this pandemic. Thus, prompt identification and intervention of kidney dysfunction is necessary during treatment.

The prevalence of initial hepatic dysfunction is also high in COVID-19, but overt liver failure as the cause of death rarely occurs (30). Liver injury is related to the hyper-inflammatory status (31, 32) instead of direct cytopathic effect (33, 34). Consistent with prior data (35, 36), we identified liver function indices, DBIL and PTA, as independent predictors for mortality, which reflects the immune dysregulation status from the perspective of hepatology. Hence, more attention should be paid toward immune dysfunction control than liver protecting therapy when dealing with liver injury (37).

The coagulopathy of COVID-19 is essentially an endothelial disease induced by cytokine storm, which contributes to the formation of hypercoagulable status and subsequent multi-organ ischemic/hemorrhagic complications in the late stage of COVID-19 (38). During this process, IL-6 facilitates clot formation by promoting the synthesis of coagulation factors and inhibiting the endogenous fibrinolytic system (39). In agreement with prior data (35, 36, 40), a coagulation marker PTA was proven to be associated with fatal outcome herein. Additionally, pre-existing CVD was also identified to be predictive of fatality. One possible explanation is that CVD usually reflects a condition of endothelial and hemorheological disorder, rendering patients more prone to negative vascular events (41). Thus, personalized medication in consideration of comorbidities should be advocated to minimize the occurrence of complications.

There are several limitations in this study. First, the IL-6 prognostic model was constructed based on all 3,342 patients in the cohort, which was validated in an internal cohort including all patients with severe disease. Additionally, we also randomly split the data into a training cohort (*N* = 1,678) and a validation cohort (*N* = 1,664). An IL-6 prognostic model was still finalized according to the methods described herein, with a high discrimination in both the training (AUC = 0.948) and validation (AUC = 0.961) cohorts (data not shown). However, external and prospective validations of this model are urgently needed. Second, since all participants were from Wuhan in the early days of the outbreak, the findings may not be generalized to other regions with diverse epidemiological characteristics worldwide. Third, this study only focused on the implications of laboratory tests in the prognosis of COVID-19, while other factors, such as the heterogeneities of admission time, therapeutic strategy, and medical treatment level in different hospitals, should not be ignored. Fourth, due to the limits of medical resources, not every item in laboratory tests was performed, especially in those with mild or moderate illness. The existence of missing data would inevitably contribute bias to our findings. Notwithstanding this, each laboratory variable still has results from at least 1,500 individuals, which we feel is sufficient for statistical analysis. Last but not least, despite the inclusion of broad laboratory variables, as we delve deeper in understanding COVID-19, more valid laboratory tests will emerge or even replace those we found herein.

In summary, our retrospective study suggests that laboratory findings have the potential for disease grading and survival prediction in COVID-19. A prognostic nomogram based on IL-6 highlights the key role of cytokines in COVID-19 pathophysiology. Our findings shed new light on the understanding and management of this pandemic.

## Data Availability Statement

The original contributions presented in the study are included in the article/Supplementary Material, further inquiries can be directed to the corresponding authors.

## Ethics Statement

This study was approved by the National Health Commission of China and the Institutional Review Board in these hospitals. Written informed consent was waived by the ethics committee of the designated hospitals for patients with emerging infectious diseases.

## Author Contributions

LL, GL, and FW had full access to all of the data in the study and took responsibility for the integrity of the data and the accuracy of the data analysis. YB, EW, LL, and GL conceptualized the article. YZhu, YZha, SZ, YB, LC, and HL collected the data. YB, EW, SZ, and JL analyzed the data. YB and FW co-wrote the manuscript with all authors providing critical feedback and edits to the subsequent revisions. All authors approved the final draft of the manuscript.

## Conflict of Interest

The authors declare that the research was conducted in the absence of any commercial or financial relationships that could be construed as a potential conflict of interest.
