# Supplementary material for: Implications of Laboratory Tests in Disease Grading and Death Risk Stratification of COVID-19: A Retrospective Study in Wuhan, China
Source: Front Med (Lausanne). 2021 Feb 19;8:629296. doi: 10.3389/fmed.2021.629296 (PMC7938237; doi:10.3389/fmed.2021.629296)
Supplement: Supplementary file 1 [file Table_1.DOCX]

**Supplemental Figure 1. Multi-organ injury on admission in patients with COVID-19.**


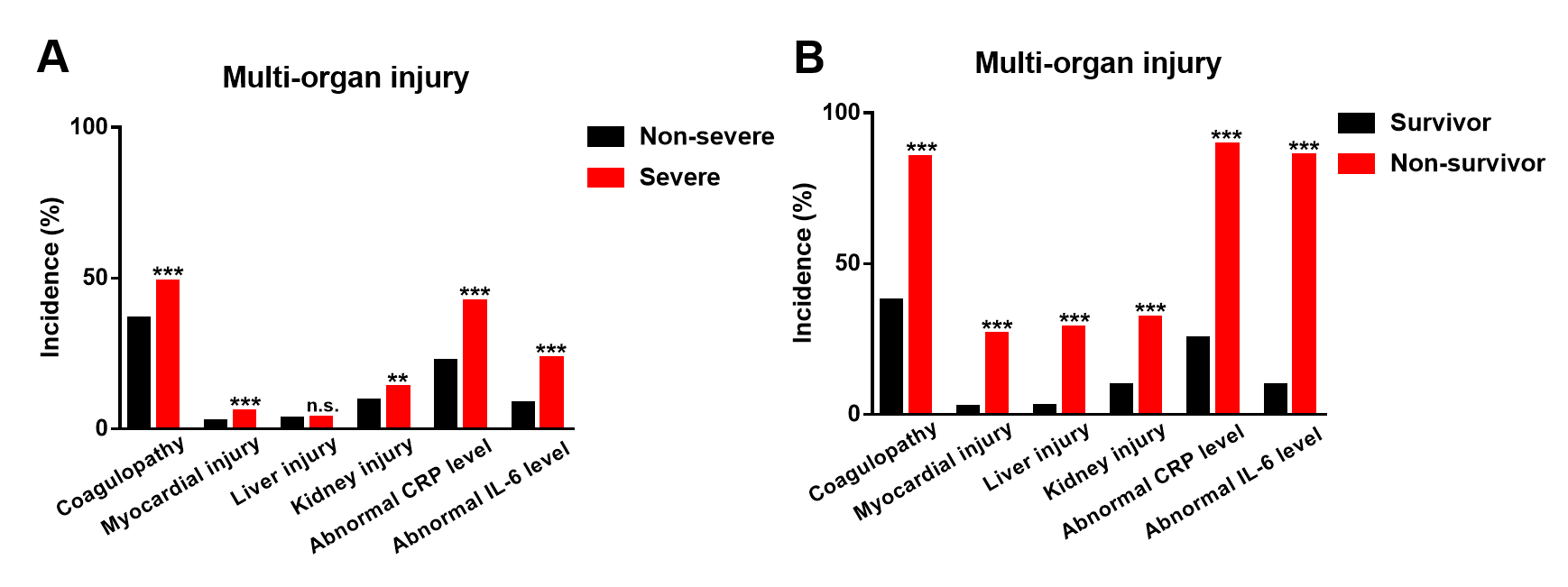


A-B, Comparison of multi-organ injury between non-severe and severe patients (A), as well as between survivors and non-survivors (B). The diagnosis of coagulopathy was made when any of the following criteria was met: PLT < 100 x 10^9^/L, D-Dimer > 1.0 mg/L, and the prolongation of PT more than 3 seconds ([1](#_ENREF_1)). Myocardial injury was suggested by serum CK-MB levels above the upper limit of the normal range ([2](#_ENREF_2)). The diagnosis of liver injury was made when any of the following criteria was met: TBIL > 51.3 μmol/L, increased serum ALT levels of at least 5 times the upper limit of the normal range, and increased serum ALP levels of at least twice the upper limit of the normal range ([3](#_ENREF_3)). Kidney injury was defined with increased serum creatinine levels of more than 1.5 times the baseline ([4](#_ENREF_4))[[4](#_ENREF_4)]. The abnormal serum levels of CRP and IL-6 were defined with an increase of at least twice the upper limit of the normal range. Abbreviations: ALP, alkaline phosphatase; ALT, alanine transaminase; CKMB, creative kinase MB; CRP, C-reactive protein; IL-6, interleukin-6; PLT, blood platelet; PT, prothrombin time; TBIL, [total](file:///D:\%E6%9C%89%E9%81%93%E8%AF%8D%E5%85%B8\Dict\7.5.2.0\resultui\dict\?keyword=total)[bilirubin](file:///D:\%E6%9C%89%E9%81%93%E8%AF%8D%E5%85%B8\Dict\7.5.2.0\resultui\dict\?keyword=bilirubin). ** *P* < 0.01, *** *P* < 0.001, n.s., not significant.

**Supplemental Figure 2: Calibration curves of the nomogram based on IL-6 for predicting survival probability.**


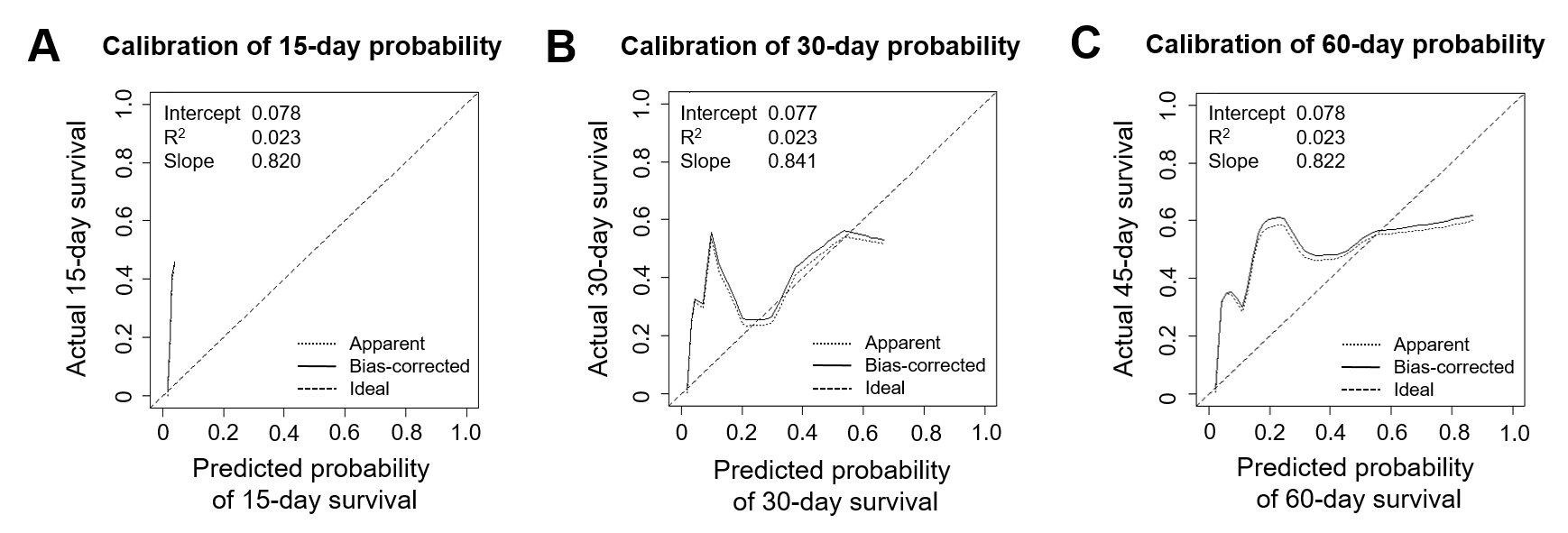


A-C, Calibration curves of the nomogram predicting 15-day (A), 30-day (B), and 60-day (C) survival probabilities in patients with COVID-19. The values of intercept, R^2^, and slope were from the corrected indices.

**Supplemental Figure 3: Kaplan-Meier survival plots for different prognostic factors of patients in severe group.**


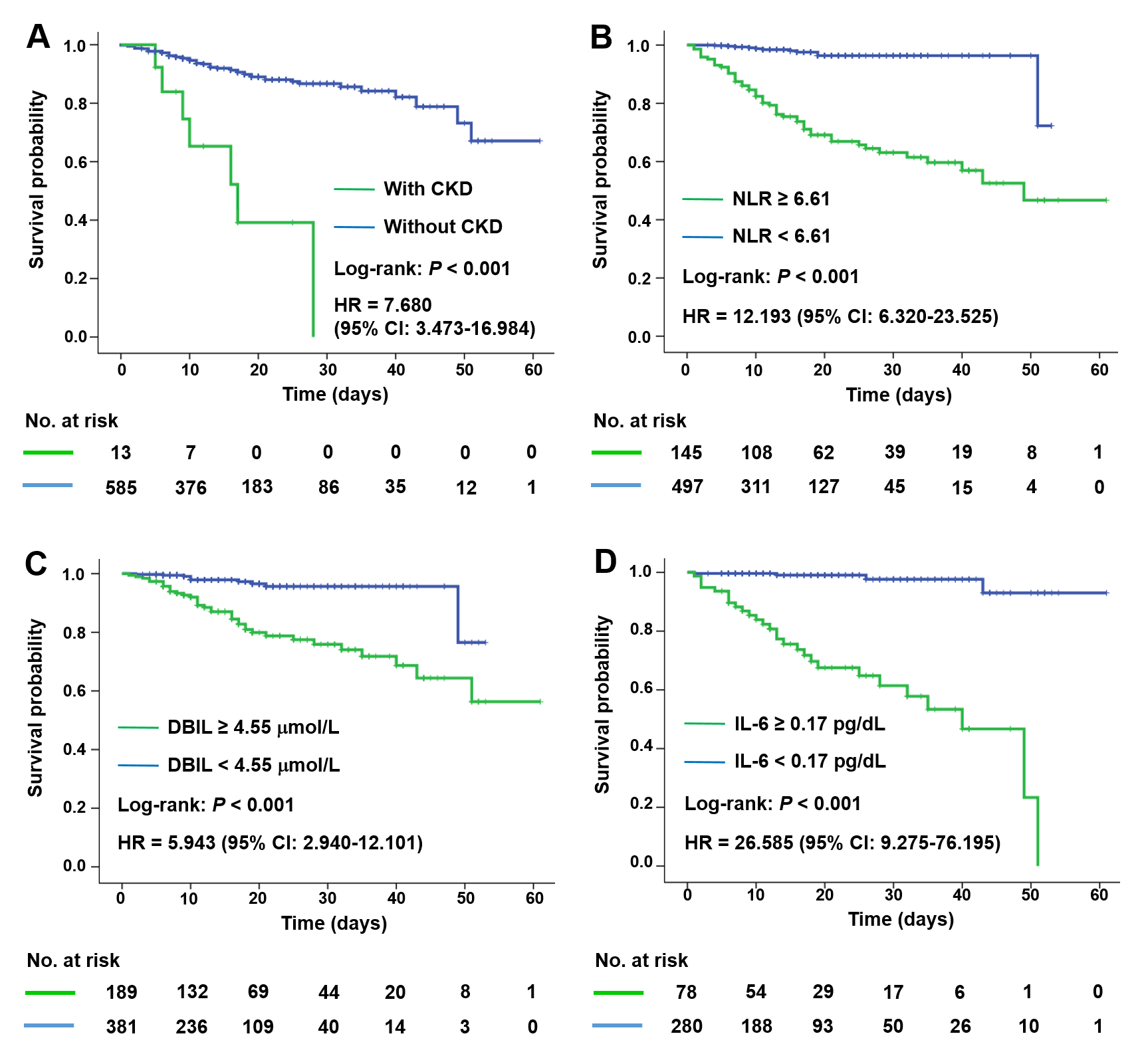


Mortality was significantly higher in patients with CKD (A), NLR ≥ 6.61 (B), DBIL ≥ 4.55 μmol/L (C), and IL-6 ≥ 0.17 pg/dL (D) according to corresponding ROC cut-offs. Abbreviations: CKD, chronic kidney disease; DBIL, direct bilirubin; HR, hazard ratio; IL-6, interleukin-6; NLR, neutrophil-to-lymphocyte ratio.

**Supplemental Table 1. Clinical characteristics and laboratory results of patients with COVID-19**

|  | **Total** | **Severe group** | **Non-severe group** | ***P* value ^a^** | **Non-survivor** | **Survivor** | ***P* value ^b^** | |
| --- | --- | --- | --- | --- | --- | --- | --- | --- |
| **Variable** | **(n = 3342)** | **(n = 648)** | **(n = 2694)** |  | **(n = 100)** | **(n = 3242)** |  | |
| **Age, y** | 61 (50-69) | 66 (57-74) | 59 (49-67) | <.001 | 75 (66-81) | 61 (50-68) | <.001 |  |
| **Male, No. (%)** | 1684 (50.39) | 353 (54.48) | 1331 (49.41) | 0.020 | 64 (64.00) | 1620 (48.47) | 0.006 |  |
| **Comorbidity, No. (%)** |  |  |  |  |  |  |  |  |
| Hypertension | 892 (26.69) | 228 (35.19) | 664 (24.65) | <.001 | 51 (51.00) | 841 (25.16) | <.001 |  |
| Diabetes | 412 (12.33) | 111 (17.13) | 301 (11.17) | <.001 | 28 (28.00) | 384 (11.49) | <.001 |  |
| CHD | 207 (6.19) | 73 (11.27) | 134 (4.97) | <.001 | 24 (24.00) | 183 (5.48) | <.001 |  |
| CVD **^c^** | 115 (3.44) | 42 (6.48) | 73 (2.71) | <.001 | 17 (17.00) | 98 (2.93) | <.001 |  |
| AF | 44 (1.32) | 22 (3.40) | 22 (0.82) | <.001 | 14 (14.00) | 30 (0.90) | <.001 |  |
| COPD | 108 (3.23) | 40 (6.17) | 68 (2.52) | <.001 | 10 (10.00) | 98 (2.93) | 0.002 |  |
| CKD **^d^** | 38 (1.14) | 13 (2.01) | 25 (0.93) | 0.021 | 12 (12.00) | 26 (0.78) | <.001 |  |
| Malignancy | 92 (2.75) | 27 (4.17) | 65 (2.41) | 0.016 | 9 (9.00) | 83 (2.48) | 0.003 |  |
| **Blood routine test, median (IQR)** | | | | | | | |  |
| RBC, ×10^12^/L (3.8-5.1) | 4.02 (3.68-4.37) | 3.92 (3.54-4.25) | 4.05 (3.71-4.40) | <.001 | 3.93 (3.34-4.32) | 4.02 (3.68-4.38) | 0.008 |  |
| Hb, g/L (115-150) | 124 (113-135) | 121 (109-133) | 125 (114-136) | <.001 | 117 (100-132.5) | 124 (114-136) | 0.001 |  |
| WBC, ×10^9^/L (3.5-9.5) | 5.7 (4.7-7.0) | 6.0 (4.8-7.7) | 5.7 (4.6-7.0) | <.001 | 8.2 (79.3-216.5) | 5.7 (4.6-7.0) | <.001 |  |
| NEU, ×10^9^/L (1.8-6.3) | 3.50 (2.71-4.66) | 3.97 (2.85-5.72) | 3.42 (2.67-4.48) | <.001 | 7.18 (4.89-10.75) | 3.47 (2.70-4.55) | <.001 |  |
| LYM, ×10^7^/L (110-320) | 150 (110-187) | 127 (83-169) | 155 (118-190) | <.001 | 67 (41-85) | 152 (113-189) | <.001 |  |
| MONO, ×10^7^/L (10-60) | 42 (34-54) | 43 (34-57) | 42 (34-54) | 0.430 | 35 (22-48) | 43 (34-54) | <.001 |  |
| EOS, ×10^7^/L (2-52) | 11 (6-18) | 9 (4-18) | 11 (6-18) | <.001 | 2 (0-6) | 11 (6-18) | <.001 |  |
| BASO, ×10^7^/L (0-6) | 2 (1-3) | 2 (1-3) | 2 (1-3) | <.001 | 1 (0-2) | 2 (1-3) | <.001 |  |
| NLR, (1.8-5.7) | 2.31 (1.69-3.40) | 3.02 (1.98-5.87) | 2.18 (1.65-3.09) | <.001 | 11.84 (6.79-21.01) | 2.27 (1.68-3.26) | <.001 |  |
| PLT, ×10^9^/L (125-350) | 223 (180-273) | 218 (166-269) | 224 (183-274) | 0.001 | 151 (79-217) | 224 (183-274) | <.001 |  |

**Supplemental Table 1. Clinical characteristics and laboratory results of patients with COVID-19 (continued)**

|  | **Total** | **Severe group** | **Non-severe group** | ***P* value ^a^** | **Non-survivor** | **Survivor** | ***P* value ^b^** | |
| --- | --- | --- | --- | --- | --- | --- | --- | --- |
| **Variable** | **(n = 3342)** | **(n = 648)** | **(n = 2694)** |  | **(n = 100)** | **(n = 3242)** |  | |
| **Coagulation function test, median (IQR)** | | | | | | | |  |
| FIB, g/L (1.8-3.5) | 2.97 (2.62-3.40) | 3.10 (2.70-3.55) | 2.95 (2.61-3.35) | <.001 | 3.05 (2.31-4.20) | 2.97 (2.63-3.39) | 0.333 |  |
| APTT, s (21-37) | 28.21 (26.31-30.41) | 28.36 (26.03-30.54) | 28.18 (26.35-30.37) | 0.824 | 31.00 (27.70-35.50) | 28.15 (26.30-30.31) | <.001 |  |
| PT, s (9.2-15) | 12.75 (12.11-13.50) | 12.92 (12.20-13.85) | 12.71 (12.08-13.42) | <.001 | 15.32 (12.75-16.38) | 12.72 (12.10-13.45) | <.001 |  |
| TT, s (14-21) | 15.21 (14.50-16.03) | 15.41 (14.59-16.38) | 15.16 (14.47-15.92) | <.001 | 17.33 (15.98-19.84) | 15.17 (14.49-15.96) | <.001 |  |
| INR, (0.8-1.25) | 1.06 (1.01-1.13) | 1.08 (1.02-1.16) | 1.06 (1.01-1.12) | <.001 | 1.28 (1.13-1.42) | 1.06 (1.01-1.12) | <.001 |  |
| D-Dimer, mg/L (0-0.55) | 0.42 (0.22-1.13) | 0.75 (0.38-1.71) | 0.38 (0.19-0.72) | <.001 | 3.52 (1.02-7.46) | 0.41 (0.21-0.84) | <.001 |  |
| PTA, (70-125) | 95.7 (92.1-99.0) | 94.8 (90.3-98.5) | 95.9 (92.5-99.1) | <.001 | 82.9 (75.7-90.6) | 95.9 (92.4-99.1) | <.001 |  |
| **Myocardial injury markers, median (IQR)** | | | | | | | |  |
| CK, IU/L (24-170) | 48.4 (34.3-71.6) | 46.3 (32.6-72.4) | 49.0 (34.8-71.4) | 0.331 | 108.3 (46.3-192.1) | 47.9 (34.2-70.0) | <.001 |  |
| CKMB, IU/L (0-24) | 8.3 (6.6-10.9) | 9.0 (7.0-12.9) | 8.2 (6.5-10.5) | <.001 | 17.1 (10.4-26.3) | 8.3 (6.6-10.6) | <.001 |  |
| LDH, IU/L (120-250) | 178.0 (151.7-217.5) | 202.0 (166.4-269.2) | 173.6 (149.9-207.4) | <.001 | 445.4 (329.2-617.1) | 176.6 (151.2-213.8) | <.001 |  |
| α-HBDH, IU/L (72-182) | 144.7 (123.9-177.9) | 164.4 (134.6-227.5) | 140.9 (121.9-170.2) | <.001 | 360.7 (271.5-529.9) | 143.3 (123.4-175.1) | <.001 |  |
| **Liver function indices, median (IQR)** | | | | | | | |  |
| ALT, IU/L (7-40) | 22.6 (14.5-37.6) | 21.9 (14.2-37.9) | 22.8 (14.6-37.5) | 0.577 | 25.0 (15.0-49.1) | 22.6 (14.5-37.4) | 0.136 |  |
| AST, IU/L (7-45) | 19.55 (15.44-26.70) | 20.80 (16.20-31.43) | 19.30 (15.30-26.1) | <.001 | 31.85 (21.47-49.17) | 19.40 (15.40-26.30) | <.001 |  |
| ALB, g/L (40-55) | 37.8 (34.7-40.0) | 36.0 (31.8-39.0) | 38.2 (35.3-40.6) | <.001 | 31.2 (27.5-34.0) | 37.9 (35.0-40.5) | <.001 |  |
| GLB, g/L (20-30) | 27.0 (24.6-29.7) | 27.3 (24.7-30.5) | 26.9 (24.6-29.5) | 0.033 | 28.1 (25.2-31.3) | 26.9 (24.6-29.6) | 0.027 |  |
| TBIL, μmol/L (0-21) | 9.5 (7.3-12.5) | 10.1 (7.7-13.5) | 9.4 (7.3-12.3) | <.001 | 13.5 (10.2-20.7) | 9.4 (7.3-12.3) | <.001 |  |
| DBIL, μmol/L (0-8) | 3.3 (2.5-4.5) | 3.6 (2.7-5.4) | 3.3 (2.4-4.3) | <.001 | 6.5 (4.3-9.2) | 3.3 (2.5-4.4) | <.001 |  |
| IBIL, μmol/L (3.4-17) | 6.1 (4.6-8.0) | 6.2 (4.7-8.1) | 6.1 (4.6-8) | 0.325 | 7.3 (4.9-10.0) | 6.1 (4.6-8.0) | 0.012 |  |

**Supplemental Table 1. Clinical characteristics and laboratory results of patients with COVID-19 (continued)**

|  | **Total** | **Severe group** | **Non-severe group** | ***P* value ^a^** | **Non-survivor** | **Survivor** | ***P* value ^b^** | |
| --- | --- | --- | --- | --- | --- | --- | --- | --- |
| **Variable** | **(n = 3342)** | **(n = 648)** | **(n = 2694)** |  | **(n = 100)** | **(n = 3242)** |  | |
| **Liver function indices, median (IQR)** | | | | | | | |  |
| TBA, μmol/L (0-10) | 3.9 (2.5-6.2) | 4 (2.6-6.3) | 3.9 (2.5-6.2) | 0.367 | 4.3 (2.7-7.0) | 3.9 (2.5-6.2) | 0.269 |  |
| ALP, IU/L (35-135) | 70.8 (58.8-85.3) | 73.4 (61.0-92.3) | 70.0 (58.3-84.1) | <.001 | 93.4 (69.6-114.2) | 70.5 (58.7-84.8) | <.001 |  |
| γ-GT, IU/L (7-45) | 30.0 (20.1-49.1) | 33.4 (23.2-54.6) | 29.0 (19.4-48.1) | <.001 | 40.6 (27.0-95.1) | 29.8 (20.0-48.5) | 0.001 |  |
| **Kidney function indices, median (IQR)** | | | | | | | |  |
| BUN, mmol/L (3.1-8.8) | 4.40 (3.62-5.72) | 4.69 (3.79-6.20) | 4.34 (3.60-5.39) | <.001 | 8.63 (5.57-12.75) | 4.38 (3.61-5.42) | <.001 |  |
| Cr, μmol/L (41-81) | 64.7 (55.3-76.1) | 65.4 (54.5-77.9) | 64.5 (55.5-75.5) | 0.512 | 78.0 (60.8-107.9) | 64.4 (55.2-75.7) | <.001 |  |
| UA, μmol/L (142-340) | 277 (222-338) | 269 (209-343) | 281 (227-338) | 0.005 | 266 (202-369) | 278 (224-338) | 0.382 |  |
| CysC, mg/L (22-29) | 0.93 (0.82-1.07) | 0.98 (0.86-1.15) | 0.92 (0.82-1.05) | <.001 | 1.19 (1.04-1.53) | 0.92 (0.82-1.06) | <.001 |  |
| **Infection-related indices, median (IQR)** | | | | | | | |  |
| CRP, mg/L (0-4) | 2.25 (0.78-8.85) | 4.83 (1.31-26.58) | 1.99 (0.72-6.36) | <.001 | 78.43 (21.61-134.76) | 2.18 (0.76-7.77) | <.001 |  |
| hsCRP, mg/L (0-4) | 2.23 (0.79-8.69) | 5.31 (1.43-10.00) | 1.93 (0.71-6.15) | <.001 | 10.00 (10.00-21.47) | 2.13 (0.76-7.48) | <.001 |  |
| IL-6, pg/dL (< 0.07) | 0.020 (0.015-0.049) | 0.039 (0.015-0.125) | 0.017 (0.015-0.038) | <.001 | 0.619 (0.269-2.673) | 0.020 (0.015-0.043) | <.001 |  |
| **Electrolytes and glucose, median (IQR)** | | | | | | | |  |
| CO_2_, mmol/L (22-29) | 24.3 (22.9-25.8) | 24.1 (22.6-25.8) | 24.3 (22.9-25.8) | 0.062 | 22.2 (19.4-25.3) | 24.3 (22.9-25.8) | <.001 |  |
| Na^+^, mmol/L (137-147) | 141.6 (139.9-143.2) | 141.1 (139.1-143.1) | 141.6 (140.0-143.2) | <.001 | 140.0 (136.5-145.2) | 141.6 (139.9-143.1) | 0.036 |  |
| K^+^, mmol/L (3.5-5.3) | 4.25 (3.96-4.55) | 4.21 (3.91-4.50) | 4.26 (3.97-4.55) | 0.007 | 4.27 (3.80-4.60) | 4.25 (3.96-4.54) | 0.931 |  |
| Ca^2+^, mmol/L (211-252) | 216 (208-223) | 213 (202-221) | 217 (209-224) | <.001 | 197 (187-204) | 216 (209-223) | <.001 |  |
| Cl^-^, mmol/L (99-110) | 106.0 (104.0-107.0) | 105.8 (103.3-107.7) | 106.1 (104.1-107.8) | 0.028 | 104.8 (100.1-110.1) | 106.0 (104.0-107.7) | 0.141 |  |
| Glu, mmol/L (3.9-6.1) | 4.89 (4.47-5.72) | 5.25 (4.61-6.44) | 4.84 (4.44-5.56) | <.001 | 7.70 (5.68-9.76) | 4.87 (4.47-5.65) | <.001 |  |

SI conversion factors: To convert LYM to ×10^9^/L, multiply by .01; MONO to ×10^9^/L, multiply by .01; EOS to ×10^9^/L, multiply by .01; BASO to ×10^9^/L, multiply by .01; D-Dimer to nmol/L, multiply by 5.486; IL-6 to pg/mL, multiply by 100.

Abbreviations: AF, atrial fibrillation; ALB, albumin; ALP, alkaline phosphatase; ALT, alanine transaminase; APTT, activated partial thromboplastin time; AST, aspartate transaminase; BASO, basophil; BUN, blood urea nitrogen; CHD, coronary heart disease; CK, creatine kinase; CKD, chronic kidney disease; CKMB, creative kinase MB; COPD, chronic obstructive pulmonary disease; Cr, creatinine; CRP, C-reactive protein; CVD, cerebrovascular disease; CysC, [cystatin](file:///D:\%E6%9C%89%E9%81%93%E8%AF%8D%E5%85%B8\Dict\7.5.2.0\resultui\dict\?keyword=cystatin)[C](file:///D:\%E6%9C%89%E9%81%93%E8%AF%8D%E5%85%B8\Dict\7.5.2.0\resultui\dict\?keyword=C); DBIL, direct bilirubin; EOS, eosinophil; FIB, fibrinogen; GLB, globulin; Glu, glucose; γ-GT, glutamyl transpeptidase; Hb, hemoglobin; α-HBDH, α-hydroxybutyrate dehydrogenase; hsCRP, high-sensitivity C-reactive protein; IBIL, indirect bilirubin; IL-6, interleukin-6; INR, international normalized ratio; LDH, lactate dehydrogenase; LYM, lymphocyte; MONO, monocyte; NEU, neutrophil; NLR, neutrophil-to-lymphocyte ratio; PLT, blood platelet; PT, prothrombin time; PTA, prothrombin activity; RBC, red blood cell; TBIL, [total](file:///D:\%E6%9C%89%E9%81%93%E8%AF%8D%E5%85%B8\Dict\7.5.2.0\resultui\dict\?keyword=total)[bilirubin](file:///D:\%E6%9C%89%E9%81%93%E8%AF%8D%E5%85%B8\Dict\7.5.2.0\resultui\dict\?keyword=bilirubin); TBA, total bile acid; TT, thrombin time; UA, [uric acid](javascript:;); WBC, white blood cell.

^a^ Compared between severe and non-severe groups with COVID-19.

^b^ Compared between non-survivors and survivors.

^c^ Including cerebral infarction and intracerebral hemorrhage.

^d^ Including primary glomerular disease, diabetic nehprophathy, hypertensive nephrosclerosis, lupus nephropathy, as well as chronic renal failure of varying etiology.

**Supplemental Table 2. Univariate logistic regression and receiver operating characteristic analyses on the risk factors for severe group**

| **Variable** | **Logistic regression analysis** | | **ROC analysis** | | | |
| --- | --- | --- | --- | --- | --- | --- |
|  | **OR (95% CI)** | ***P* value** | **AUC (95% CI)** | ***P* value** | **Sensitivity** | **Specificity** |
| **Age, y** | 1.042 (1.035-1.050) | <.001 | 0.657 (0.634-0.680) | <.001 | 61.0% | 61.6% |
| **Male** | 1.225 (1.032-1.455) | 0.021 | 0.525 (0.501-0.550) | 0.045 | 54.5% | 50.6% |
| **Comorbidity** | | | | | | |
| Hypertension | 1.669 (1.384-2.013) | <.001 | 0.556 (0.530-0.582) | <.001 | 38.1% | 73.1% |
| Diabetes | 1.640 (1.292-2.083) | <.001 | 0.532 (0.505-0.558) | 0.016 | 18.6% | 87.8% |
| CHD | 2.421 (1.793-3.269) | <.001 | 0.534 (0.507-0.560) | 0.010 | 12.2% | 94.6% |
| AF | 4.245 (2.335-7.719) | <.001 | 0.514 (0.488-0.540) | 0.290 | 3.7% | 99.1% |
| CVD | 2.477 (1.676-3.661) | <.001 | 0.520 (0.494-0.547) | 0.123 | 7.0% | 97.0% |
| COPD | 2.529 (1.693-3.778) | <.001 | 0.520 (0.493-0.546) | 0.135 | 6.7% | 97.2% |
| CKD | 2.171 (1.104-4.269) | 0.025 | 0.506 (0.480-0.532) | 0.659 | 2.2% | 99.0% |
| Malignancy | 1.744 (1.103-2.757) | 0.017 | 0.509 (0.483-0.535) | 0.476 | 4.5% | 97.4% |
| **Blood routine test** | | | | | | |
| RBC, ×10^12^/L | 0.579 (0.493-0.680) | <.001 | 0.579 (0.553-0.604) | <.001 | 61.0% | 51.4% |
| Hb, g/L | 0.984 (0.979-0.989) | <.001 | 0.571 (0.545-0.598) | <.001 | 52.0% | 59.3% |
| WBC, ×10^9^/L | 1.113 (1.076-1.151) | <.001 | 0.556 (0.528-0.583) | <.001 | 26.8% | 82.2% |
| NEU, ×10^9^/L | 1.173 (1.133-1.214) | <.001 | 0.594 (0.568-0.620) | <.001 | 54.6% | 60.6% |
| LYM, ×10^7^/L | 0.994 (0.992-0.995) | <.001 | 0.632 (0.606-0.657) | <.001 | 34.6% | 86.2% |
| MONO, ×10^7^/L | 1.003 (0.998-1.007) | 0.274 |  |  |  |  |
| EOS, ×10^7^/L | 0.993 (0.985-1.001) | 0.069 |  |  |  |  |
| BASO, ×10^7^/L | 0.882 (0.828-0.940) | <.001 | 0.558 (0.531-0.586) | <.001 | 43.2% | 68.0% |
| NLR | 1.105 (0.083-1.128) | <.001 | 0.649 (0.623-0.674) | <.001 | 48.0% | 76.9% |
| PLT, ×10^9^/L | 0.998 (0.997-0.999) | <.001 | 0.542 (0.516-0.568) | 0.001 | 26.8% | 82.2% |
| **Coagulation function test** | | | | | | |
| FIB, g/L | 1.172 (1.043-1.317) | 0.008 | 0.568 (0.540-0.596) | <.001 | 36.9% | 75.2% |
| APTT, s | 1.020 (1.004-1.036) | 0.013 | 0.503 (0.476-0.530) | 0.824 | 13.1% | 90.8% |
| PT, s | 1.081 (1.037-1.127) | <.001 | 0.561 (0.534-0.588) | <.001 | 37.4% | 74.6% |
| TT, s | 1.181 (1.113-1.253) | <.001 | 0.573 (0.546-0.601) | <.001 | 44.8% | 67.3% |
| INR | 2.659 (1.594-4.435) | <.001 | 0.565 (0.538-0.592) | <.001 | 38.5% | 73.4% |
| D-Dimer, mg/L | 1.075 (1.038-1.113) | <.001 | 0.685 (0.660-0.709) | <.001 | 71.2% | 57.5% |
| PTA | 0.964 (0.952-0.976) | <.001 | 0.569 (0.540-0.597) | <.001 | 39.9% | 73.6% |
| **Myocardial injury markers** | | | | | | |
| CK, IU/L | 1.003 (1.002-1.004) | <.001 | 0.513 (0.485-0.541) | 0.331 | 32.6% | 73.7% |
| CKMB, IU/L | 1.021 (1.012-1.030) | <.001 | 0.577 (0.551-0.602) | <.001 | 25.1% | 87.1% |
| LDH, IU/L | 1.006 (1.005-1.007) | <.001 | 0.649 (0.623-0.675) | <.001 | 54.6% | 68.4% |
| α-HBDH, IU/L | 1.007 (1.006-1.008) | <.001 | 0.650 (0.624-0.676) | <.001 | 45.5% | 77.5% |

**Supplemental Table 2. Univariate logistic regression and receiver operating characteristic analyses on the risk factors for severe group (continued)**

| **Variable** | **Logistic regression analysis** | | **ROC analysis** | | | |
| --- | --- | --- | --- | --- | --- | --- |
|  | **OR (95% CI)** | ***P* value** | **AUC (95% CI)** | ***P* value** | **Sensitivity** | **Specificity** |
| **Liver function indices** | | | | | | |
| ALT, IU/L | 1.000 (0.997-1.002) | 0.892 |  |  |  |  |
| AST, IU/L | 1.007 (1.003-1.011) | <.001 | 0.555 (0.530-0.581) | <.001 | 27.0% | 82.1% |
| ALB, g/L | 0.891 (0.874-0.909) | <.001 | 0.640 (0.615-0.664) | <.001 | 44.9% | 75.6% |
| GLB, g/L | 1.031 (1.009-1.053) | 0.005 | 0.529 (0.501-0.556) | 0.032 | 39.5% | 67.3% |
| TBIL, μmol/L | 1.032 (0.019-1.046) | <.001 | 0.550 (0.525-0.576) | <.001 | 62.6% | 45.5% |
| DBIL, μmol/L | 1.077 (1.043-1.113) | <.001 | 0.575 (0.548-0.603) | <.001 | 32.1% | 79.8% |
| IBIL, μmol/L | 1.033 (1.008-1.059) | 0.009 | 0.513 (0.487-0.540) | 0.315 | 11.8% | 92.5% |
| TBA, μmol/L | 1.003 (0.993-1.013) | 0.560 |  |  |  |  |
| ALP, IU/L | 1.005 (1.002-1.007) | <.001 | 0.552 (0.526-0.577) | <.001 | 24.5% | 84.4% |
| γ-GT, IU/L | 1.002 (1.000-1.004) | 0.028 | 0.558 (0.533-0.584) | <.001 | 74.6% | 37.3% |
| **Kidney function indices** | | | | | | |
| BUN, mmol/L | 1.126 (1.090-1.164) | <.001 | 0.569 (0.542-0.595) | <.001 | 30.1% | 82.6% |
| Cr, μmol/L | 1.002 (1.000-1.004) | 0.060 |  |  |  |  |
| UA, μmol/L | 0.999 (0.998-1.000) | 0.164 |  |  |  |  |
| CysC, mg/L | 1.986 (1.576-2.504) | <.001 | 0.602 (0.575-0.628) | <.001 | 52.2% | 63.4% |
| **Infection-related indices** | | | | | | |
| CRP, mg/L | 1.012 (1.009-1.014) | <.001 | 0.633 (0.606-0.660) | <.001 | 53.1% | 68.2% |
| hsCRP, mg/L | 1.017 (1.011-1.024) | <.001 | 0.645 (0.620-0.670) | <.001 | 55.2% | 69.0% |
| IL-6, pg/dL | 1.150 (1.001-1.321) | 0.049 | 0.649 (0.615-0.683) | <.001 | 50.3% | 75.8% |
| **Electrolytes and glucose** | | | | | | |
| CO_2_, mmol/L | 0.966 (0.931-1.001) | 0.059 |  |  |  |  |
| Na^+^, mmol/L | 0.965 (0.938-0.992) | 0.013 | 0.550 (0.521-0.578) | <.001 | 41.7% | 69.5% |
| K^+^, mmol/L | 0.811 (0.675-0.976) | 0.026 | 0.536 (0.509-0.562) | 0.007 | 43.2% | 63.4% |
| Ca^2+^, mmol/L | 0.972 (0.965-0.980) | <.001 | 0.600 (0.573-0.627) | <.001 | 43.7% | 73.1% |
| Cl^-^, mmol/L | 0.974 (0.948-1.001) | 0.058 |  |  |  |  |
| Glu, mmol/L | 1.095 (1.060-1.131) | <.001 | 0.596 (0.571-0.622) | <.001 | 50.2% | 67.8% |

Abbreviations: AF, atrial fibrillation; ALB, albumin; ALP, alkaline phosphatase; ALT, alanine transaminase; APTT, activated partial thromboplastin time; AST, aspartate transaminase; AUC, area under the curve; BASO, basophil; BUN, blood urea nitrogen; CHD, coronary heart disease; CK, creatine kinase; CKD, chronic kidney disease; CKMB, creative kinase MB; COPD, chronic obstructive pulmonary disease; Cr, creatinine; CRP, C-reactive protein; CVD, cerebrovascular disease; CysC, [cystatin](file:///D:\%E6%9C%89%E9%81%93%E8%AF%8D%E5%85%B8\Dict\7.5.2.0\resultui\dict\?keyword=cystatin)[C](file:///D:\%E6%9C%89%E9%81%93%E8%AF%8D%E5%85%B8\Dict\7.5.2.0\resultui\dict\?keyword=C); DBIL, direct bilirubin; EOS, eosinophil; FIB, fibrinogen; GLB, globulin; Glu, glucose; γ-GT, glutamyl transpeptidase; Hb, hemoglobin; α-HBDH, α-hydroxybutyrate dehydrogenase; hsCRP, high-sensitivity C-reactive protein; IBIL, indirect bilirubin; IL-6, interleukin-6; INR, international normalized ratio; LDH, lactate dehydrogenase; LYM, lymphocyte; MONO, monocyte; NEU, neutrophil; NLR, neutrophil-to-lymphocyte ratio; OR, odds ratio; PLT, blood platelet; PT, prothrombin time; PTA, prothrombin activity; RBC, red blood cell; ROC, receiver operating characteristic curve; TBIL, [total](file:///D:\%E6%9C%89%E9%81%93%E8%AF%8D%E5%85%B8\Dict\7.5.2.0\resultui\dict\?keyword=total)[bilirubin](file:///D:\%E6%9C%89%E9%81%93%E8%AF%8D%E5%85%B8\Dict\7.5.2.0\resultui\dict\?keyword=bilirubin); TBA, total bile acid; TT, thrombin time; UA, [uric acid](javascript:;); WBC, white blood cell.

**Supplemental Table 3. Multivariate logistic regression analysis on the risk factors for severe group**

| **Variable** | **OR (95% CI)** | ***P* value** |
| --- | --- | --- |
| Age, y | 1.032 (1.021-1.043) | <.001 |
| NLR | 1.090 (1.045-1.137) | <.001 |
| α-HBDH, IU/L | 1.004 (1.002-1.006) | 0.001 |

Abbreviations: AF, atrial fibrillation; AUC, area under the curve; BUN, blood urea nitrogen; CHD, coronary heart disease; CKD, chronic kidney disease; COPD, chronic obstructive pulmonary disease; CVD, cerebrovascular disease; CysC, [cystatin](file:///D:\%E6%9C%89%E9%81%93%E8%AF%8D%E5%85%B8\Dict\7.5.2.0\resultui\dict\?keyword=cystatin)[C](file:///D:\%E6%9C%89%E9%81%93%E8%AF%8D%E5%85%B8\Dict\7.5.2.0\resultui\dict\?keyword=C); DBIL, direct bilirubin; Glu, glucose;α-HBDH, α-hydroxybutyrate dehydrogenase; hsCRP, high-sensitivity C-reactive protein; IL-6, interleukin-6; NLR, neutrophil-to-lymphocyte ratio; OR, odds ratio; TT, thrombin time.

The model contains 20 variables, including all significant demographic variables (age, sex, hypertension, diabetes, CHD, AF, CVD, COPD, CKD, malignancy) identified in the univariate analysis, as well as one or two variables with the highest AUC in each category of laboratory tests (NLR, D-Dimer, TT, α-HBDH, DBIL, BUN, CysC, hsCRP, IL-6, and Glu).

**Supplemental Table 4. Comparison of possible diagnostic models for identifying patients in severe group**

| **Models ^a^** | **AUC (95% CI)** | **LR χ2** | **df** | **HL χ2** |
| --- | --- | --- | --- | --- |
| Model 1: LP = age | 0.657 (0.634-0.680) | 162.869 | 1 | 4.783 ^b^ |
| Model 2: LP = NLR | 0.649 (0.623-0.674) | 134.471 | 1 | 38.060 |
| Model 3: LP = α-HBDH | 0.650 (0.624-0.676) | 148.239 | 1 | 15.417 ^b^ |
| Model 4: LP = 0.315 × age + 0.086 × NLR + 0.004 × α-HBDH | 0.694 (0.668-0.719) | 241.489 | 3 | 10.976 ^b^ |

Abbreviations: AUC, area under the curve; df, degree of freedom; α-HBDH, α-hydroxybutyrate dehydrogenase; HL χ2: Hosmer-Lemeshow χ2; LP: linear predictor; LR χ2: likelyhood ratio χ2; NLR, neutrophil-to-lymphocyte ratio.

^a^ The coefficients of variables in Model 4 were derived from multivariate logistic regression analysis.

**^b^** *P* value > 0.05.

If not specifically denoted, *P* values of AUCs, LR χ2 or HL χ2 in the models < 0.001.

**Supplemental Table 5. Univariate Cox regression and receiver operating characteristic analyses on the risk factors for mortality of all patients with COVID-19**

| **Variable** | **Cox Regression Analysis** | | **ROC Analysis** | | | |
| --- | --- | --- | --- | --- | --- | --- |
|  | **HR (95% CI)** | ***P* value** | **AUC (95% CI)** | ***P* value** | **Sensitivity** | **Specificity** |
| **Age, y** | 1.088 (1.069-1.108) | <.001 | 0.788 (0.743-0.833) | <.001 | 72.0% | 72.5% |
| **Male** | 1.690 (1.123-2.544) | 0.012 | 0.570 (0.514-0.626) | 0.017 | 64.0% | 50.0% |
| **Comorbidity** | | | | | | |
| Hypertension | 2.262 (1.522-3.360) | <.001 | 0.616 (0.557-0.675) | <.001 | 51.5% | 71.6% |
| Diabetes | 2.198 (1.417-3.409) | <.001 | 0.577 (0.515-0.639) | 0.009 | 28.3% | 87.1% |
| CHD | 4.370 (2.759-6.923) | <.001 | 0.590 (0.527-0.654) | 0.002 | 24.2% | 93.8% |
| AF | 8.492 (4.753-15.174) | <.001 | 0.566 (0.503-0.629) | 0.026 | 14.1% | 99.0% |
| CVD | 5.101 (3.018-8.622) | <.001 | 0.569 (0.506-0.632) | 0.019 | 17.2% | 96.7% |
| COPD | 2.757 (1.421-5.349) | <.001 | 0.534 (0.473-0.595) | 0.249 | 10.1% | 96.7% |
| CKD | 15.828 (8.606-29.109) | <.001 | 0.556 (0.494-0.619) | 0.057 | 12.1% | 99.1% |
| Malignancy | 3.446 (1.735-6.844) | <.001 | 0.531 (0.471-0.591) | 0.210 | 9.0% | 97.2% |
| **Blood routine test** | | | | | | |
| RBC, ×10^12^/L | 0.632 (0.447-0.895) | 0.010 | 0.581 (0.513-0.648) | 0.008 | 32.6% | 85.4% |
| Hb, g/L | 0.979 (0.968-0.990) | <.001 | 0.599 (0.532-0.665) | 0.001 | 40.4% | 80.4% |
| WBC, ×10^9^/L | 1.136 (1.110-1.163) | <.001 | 0.721 (0.651-0.792) | <.001 | 49.3% | 88.7% |
| NEU, ×10^9^/L | 1.149 (1.128-1.170) | <.001 | 0.825 (0.772-0.878) | <.001 | 78.4% | 78.3% |
| LYM, ×10^7^/L | 0.972 (0.967-0.977) | <.001 | 0.872 (0.829-0.915) | <.001 | 78.9% | 87.2% |
| MONO, ×10^7^/L | 0.986 (0.973-0.998) | 0.027 | 0.629 (0.556-0.703) | <.001 | 62.8% | 61.5% |
| EOS, ×10^7^/L | 0.903 (0.869-0.938) | <.001 | 0.802 (0.744-0.860) | <.001 | 72.9% | 83.7% |
| BASO, ×10^7^/L | 0.523 (0.420-0.651) | <.001 | 0.750 (0.695-0.806) | <.001 | 74.4% | 66.9% |
| NLR | 1.063 (1.055-1.071) | <.001 | 0.903 (0.864-0.942) | <.001 | 82.1% | 89.2% |
| PLT, ×10^9^/L | 0.985 (0.981-0.988) | <.001 | 0.745 (0.682-0.807) | <.001 | 57.6% | 84.0% |
| **Coagulation function test** | | | | | | |
| FIB, g/L | 1.092 (0.938-1.272) | 0.255 |  |  |  |  |
| APTT, s | 1.034 (1.027-1.042) | <.001 | 0.671 (0.600-0.743) | <.001 | 55.4% | 75.8% |
| PT, s | 1.069 (1.052-1.086) | <.001 | 0.767 (0.696-0.837) | <.001 | 67.9% | 83.9% |
| TT, s | 1.070 (1.057-1.083) | <.001 | 0.804 (0.742-0.867) | <.001 | 75.6% | 75.8% |
| INR | 2.166 (1.814-2.586) | <.001 | 0.804 (0.741-0.867) | <.001 | 70.4% | 84.4% |
| D-Dimer, mg/L | 1.047 (1.036-1.059) | <.001 | 0.862 (0.817-0.906) | <.001 | 84.6% | 74.6% |
| PTA | 0.935 (0.926-0.944) | <.001 | 0.842 (0.779-0.905) | <.001 | 76.9% | 83.3% |
| **Myocardial injury markers** | | | | | | |
| CK, IU/L | 1.004 (1.003-1.005) | <.001 | 0.722 (0.642-0.801) | <.001 | 68.1% | 76.4% |
| CKMB, IU/L | 1.011 (1.009-1.014) | <.001 | 0.828 (0.780-0.875) | <.001 | 72.3% | 80.1% |
| LDH, IU/L | 1.006 (1.005-1.006) | <.001 | 0.922 (0.879-0.966) | <.001 | 87.1% | 89.4% |
| α-HBDH, IU/L | 1.007 (1.006-1.008) | <.001 | 0.922 (0.880-0.964) | <.001 | 84.3% | 91.5% |

**Supplemental Table 5. Univariate Cox regression and receiver operating characteristic analyses on the risk factors for mortality of all patients with COVID-19 (continued)**

| **Variable** | **Cox Regression Analysis** | | **ROC Analysis** | | | |
| --- | --- | --- | --- | --- | --- | --- |
|  | **HR (95% CI)** | ***P* value** | **AUC (95% CI)** | ***P* value** | **Sensitivity** | **Specificity** |
| **Liver function indices** | | | | | | |
| ALT, IU/L | 1.004 (1.000-1.007) | 0.026 | 0.547 (0.480-0.614) | 0.136 | 21.2% | 90.7% |
| AST, IU/L | 1.008 (1.006-1.010) | <.001 | 0.728 (0.666-0.790) | <.001 | 62.8% | 76.4% |
| ALB, g/L | 0.786 (0.753-0.820) | <.001 | 0.848 (0.806-0.889) | <.001 | 75.8% | 80.5% |
| GLB, g/L | 1.058 (1.008-1.110) | 0.022 | 0.577 (0.505-0.649) | 0.027 | 66.2% | 52.2% |
| TBIL, μmol/L | 1.054 (1.044-1.064) | <.001 | 0.714 (0.653-0.775) | <.001 | 77.6% | 55.9% |
| DBIL, μmol/L | 1.068 (1.053-1.082) | <.001 | 0.796 (0.734-0.858) | <.001 | 73.6% | 77.4% |
| IBIL, μmol/L | 1.115 (1.083-1.147) | <.001 | 0.587 (0.512-0.661) | 0.012 | 55.6% | 64.3% |
| TBA, μmol/L | 1.012 (0.993-1.032) | 0.203 |  |  |  |  |
| ALP, IU/L | 1.007 (1.004-1.010) | <.001 | 0.679 (0.605-0.752) | <.001 | 55.9% | 79.3% |
| γ-GT, IU/L | 1.003 (1.000-1.006) | 0.063 |  |  |  |  |
| **Kidney function indices** | | | | | | |
| BUN, mmol/L | 1.207 (1.179-1.236) | <.001 | 0.816 (0.757-0.875) | <.001 | 67.9% | 86.8% |
| Cr, μmol/L | 1.006 (1.004-1.007) | <.001 | 0.665 (0.597-0.734) | <.001 | 50.0% | 79.8% |
| UA, μmol/L | 1.004 (1.001-1.006) | 0.001 | 0.530 (0.451-0.610) | 0.382 | 40.3% | 73.5% |
| CysC, mg/L | 2.230 (1.869-2.660) | <.001 | 0.785 (0.723-0.847) | <.001 | 76.6% | 71.3% |
| **Infection-related indices** | | | | | | |
| CRP, mg/L | 1.015 (1.013-1.017) | <.001 | 0.898 (0.861-0.936) | <.001 | 91.0% | 75.0% |
| hsCRP, mg/L | 1.008 (1.007-1.010) | <.001 | 0.883 (0.852-0.914) | <.001 | 88.4% | 76.1% |
| IL-6, pg/dL | 1.082 (1.055-1.110) | <.001 | 0.953 (0.934-0.972) | <.001 | 91.8% | 86.3% |
| **Electrolytes and glucose** | | | | | | |
| CO_2_, mmol/L | 0.800 (0.753-0.849) | <.001 | 0.660 (0.578-0.741) | <.001 | 50.0% | 84.3% |
| Na^+^, mmol/L | 1.024 (0.962-1.091) | 0.459 |  |  |  |  |
| K^+^, mmol/L | 1.067 (0.720-1.580) | 0.784 |  |  |  |  |
| Ca^2+^, mmol/L | 0.915 (0.900-0.931) | <.001 | 0.858 (0.803-0.914) | <.001 | 80.0% | 82.3% |
| Cl^-^, mmol/L | 1.053 (0.990-1.119) | 0.100 | 0.551 (0.456-0.647) | 0.141 | 43.7% | 85.7% |
| Glu, mmol/L | 1.161 (1.112-1.203) | <.001 | 0.817 (0.771-0.863) | <.001 | 82.8% | 72.2% |

Abbreviations: AF, atrial fibrillation; ALB, albumin; ALP, alkaline phosphatase; ALT, alanine transaminase; APTT, activated partial thromboplastin time; AST, aspartate transaminase; AUC, area under the curve; BASO, basophil; BUN, blood urea nitrogen; CHD, coronary heart disease; CK, creatine kinase; CKD, chronic kidney disease; CKMB, creative kinase MB; COPD, chronic obstructive pulmonary disease; Cr, creatinine; CRP, C-reactive protein; CVD, cerebrovascular disease; CysC, [cystatin](file:///D:\%E6%9C%89%E9%81%93%E8%AF%8D%E5%85%B8\Dict\7.5.2.0\resultui\dict\?keyword=cystatin)[C](file:///D:\%E6%9C%89%E9%81%93%E8%AF%8D%E5%85%B8\Dict\7.5.2.0\resultui\dict\?keyword=C); DBIL, direct bilirubin; EOS, eosinophil; FIB, fibrinogen; GLB, globulin; Glu, glucose; γ-GT, glutamyl transpeptidase; Hb, hemoglobin; α-HBDH, α-hydroxybutyrate dehydrogenase; HR, hazard ratio; hsCRP, high-sensitivity C-reactive protein; IBIL, indirect bilirubin; IL-6, interleukin-6; INR, international normalized ratio; LDH, lactate dehydrogenase; LYM, lymphocyte; MONO, monocyte; NEU, neutrophil; NLR, neutrophil-to-lymphocyte ratio; PLT, blood platelet; PT, prothrombin time; PTA, prothrombin activity; RBC, red blood cell; ROC, receiver operating characteristic curve; TBIL, [total](file:///D:\%E6%9C%89%E9%81%93%E8%AF%8D%E5%85%B8\Dict\7.5.2.0\resultui\dict\?keyword=total)[bilirubin](file:///D:\%E6%9C%89%E9%81%93%E8%AF%8D%E5%85%B8\Dict\7.5.2.0\resultui\dict\?keyword=bilirubin); TBA, total bile acid; TT, thrombin time; UA, [uric acid](javascript:;); WBC, white blood cell.

**Supplemental Table 6. Multivariate Cox regression analysis on the risk factors for mortality of all patients with COVID-19**

| **Variable** | **HR (95% CI)** | ***P* value** |
| --- | --- | --- |
| CVD | 6.162 (2.270-16.725) | <.001 |
| PTA | 0.912 (0.875-0.949) | <.001 |
| BUN, mmol/L | 1.207 (1.107-1.316) | <.001 |
| IL-6, pg/dL | 1.085 (1.048-1.124) | <.001 |

Abbreviations: ALB, albumin; AF, atrial fibrillation; BUN, blood urea nitrogen; CHD, coronary heart disease; CKD, chronic kidney disease; COPD, chronic obstructive pulmonary disease; CRP, C-reactive protein; CVD, cerebrovascular disease; CysC, [cystatin](file:///D:\%E6%9C%89%E9%81%93%E8%AF%8D%E5%85%B8\Dict\7.5.2.0\resultui\dict\?keyword=cystatin)[C](file:///D:\%E6%9C%89%E9%81%93%E8%AF%8D%E5%85%B8\Dict\7.5.2.0\resultui\dict\?keyword=C); DBIL, direct bilirubin; α-HBDH, α-hydroxybutyrate dehydrogenase; HR, hazard ratio; IL-6, interleukin-6; LDH, lactate dehydrogenase; NLR, neutrophil-to-lymphocyte ratio; PTA,[prothrombin activity](javascript:;).

The model contains 21 variables, including all significant demographic variables (age, sex, hypertension, diabetes, CHD, AF, CVD, COPD, CKD, malignancy) identified in the univariate analysis, as well as one or two variables with the highest AUC in each category of laboratory tests (NLR, D-Dimer, PTA, LDH, α-HBDH, ALB, DBIL, BUN, CysC, CRP, and IL-6).

**Supplemental Table 7. Comparison of possible prognostic models for mortality of all patients with COVID-19**

| **Models ^a^** | **15-d AUC (95% CI)** | **30-d AUC (95% CI)** | **60-d AUC (95% CI)** | **LR χ2** | **df** | **C-index (95% CI)** |
| --- | --- | --- | --- | --- | --- | --- |
| Model 1: LP = CVD | 0.566 (0.489-0.643) **^b^** | 0.563 (0.496-0.629) **^c^** | 0.569 (0.506-0.632) **^c^** | 25.99 | 1 | 0.572 (0.549-0.595) |
| Model 2: LP = PTA | 0.895 (0.825-0.964) | 0.849 (0.778-0.920) | 0.842 (0.779-0.905) | 119.30 | 1 | 0.852 (0.825-0.879) |
| Model 3: LP = BUN | 0.875 (0.816-0.935) | 0.816 (0.751-0.881) | 0.816 (0.757-0.875) | 83.83 | 1 | 0.859 (0.825-0.893) |
| Model 4: LP = IL-6 | 0.954 (0.935-0.973) | 0.949 (0.927-0.972) | 0.953 (0.934-0.972) | 16.21 | 1 | 0.936 (0.925-0.947) |
| Model 5: LP = 0.082 × IL-6 + 1.818 × CVD | 0.935 (0.914-0.956) | 0.931 (0.907-0.955) | 0.936 (0.916-0.956) | 38.14 | 2 | 0.921 (0.909-0.933) |
| Model 6: LP = 0.082 × IL-6 - 0.093 × PTA | 0.950 (0.879-1.000) | 0.839 (0.731-0.947) | 0.826 (0.739-0.912) | 62.79 | 2 | 0.866 (0.818-0.914) |
| Model 7: LP = 0.082 × IL-6 + 0.188 × BUN | 0.910 (0.826-0.994) | 0.862 (0.774-0.951) | 0.863 (0.789-0.936) | 80.37 | 2 | 0.885 (0.853-0.917) |
| Model 8: LP = 0.082 × IL-6 + 0.188 × BUN – 0.093 × PTA + 1.818 × CVD | 0.968 (0.931-1.000) | 0.911 (0.819-1.000) | 0.914 (0.849-0.980) | 107.10 | 4 | 0.916 (0.881-0.951) |

Abbreviations: AUC, area under the curve; BUN, blood urea nitrogen; CVD, cerebrovascular disease; df, degree of freedom; HL χ2: Hosmer-Lemeshow χ2; IL-6, interleukin-6; LP: linear predictor; LR χ2: likelyhood ratio χ2; PTA,[prothrombin activity](javascript:;).

^a^ The coefficients of variables in Models 5-8 were derived from multivariate Cox regression analysis.

**^b^** *P* value > 0.05.

**^c^** *P* value < 0.05.

If not specifically denoted, *P* values of AUCs or LR χ2 in the models < 0.001.

**Supplemental Table 8. Clinical characteristics and laboratory results of patients in severe group**

| **Variable** | **Total** | **Non-survivor** | **Survivor** | ***P* ^a^** |
| --- | --- | --- | --- | --- |
|  | **(n = 648)** | **(n = 64)** | **(n = 584)** |  |
| **Age, y** | 66 (57-74) | 77 (69-82) | 65 (57-73) | <.001 |
| **Male, No. (%)** | 353 (54.48) | 38 (59.38) | 315 (53.94) | 0.407 |
| **Comorbidity, No. (%)** |  |  |  |  |
| Hypertension | 228 (35.19) | 32 (50.00) | 196 (33.56) | 0.029 |
| Diabetes | 111 (17.13) | 17 (26.56) | 94 (16.10) | 0.069 |
| CHD | 73 (11.27) | 18 (28.13) | 55 (9.42) | <.001 |
| CVD | 42 (6.48) | 11 (17.19) | 31 (5.31) | 0.002 |
| AF | 22 (3.40) | 9 (14.06) | 13 (2.23) | <.001 |
| COPD | 40 (6.17) | 8 (12.50) | 32 (5.48) | 0.048 |
| CKD | 13 (2.01) | 7 (10.94) | 6 (1.03) | <.001 |
| Malignancy | 27 (4.17) | 7 (10.94) | 20 (3.42) | 0.018 |
| **Blood routine test, median (IQR)** | | | | |
| RBC, ×10^12^/L (3.8-5.1) | 3.92 (3.54-4.25) | 3.73 (3.30-4.28) | 3.92 (3.56-4.26) | 0.141 |
| Hb, g/L (115-150) | 121 (109-133) | 113 (97-134) | 121 (110-133) | 0.073 |
| WBC, ×10^9^/L (3.5-9.5) | 6.0 (4.8-7.7) | 7.85 (5.78-13.73) | 5.9 (4.7-7.5) | <.001 |
| NEU, ×10^9^/L (1.8-6.3) | 3.97 (2.85-5.72) | 7.25 (4.89-11.35) | 3.79 (2.79-5.24) | <.001 |
| LYM, ×10^7^/L (110-320) | 127 (83-169) | 56 (37-81) | 135 (93-172) | <.001 |
| MONO, ×10^7^/L (10-60) | 43 (34-57) | 33 (21-42) | 44 (35-58) | <.001 |
| EOS, ×10^7^/L (2-52) | 9 (4-18) | 1 (0-3) | 11 (5-19) | <.001 |
| BASO, ×10^7^/L (0-6) | 2 (1-3) | 1.00 (0.00-1.25) | 2.00 (1.00-3.00) | <.001 |
| NLR, (1.8-5.7) | 3.02 (1.98-5.87) | 13.63 (7.65-21.61) | 2.83 (1.92-4.74) | <.001 |
| PLT, ×10^9^/L (125-350) | 218 (166-269) | 116.0 (74.5-181.8) | 223.0 (176.0-274.0) | <.001 |
| **Coagulation function test, median (IQR)** | | | | |
| FIB, g/L (1.8-3.5) | 3.10 (2.70-3.55) | 2.89 (2.22-3.89) | 3.12 (2.75-3.55) | 0.074 |
| APTT, s (21-37) | 28.36 (26.03-30.54) | 32.84 (29.24-37.32) | 28.10 (25.95-30.15) | <.001 |
| PT, s (9.2-15) | 12.92 (12.20-13.85) | 15.40 (12.95-17.10) | 12.84 (12.15-13.71) | <.001 |
| TT, s (14-21) | 15.41 (14.59-16.38) | 17.67 (16.06-20.22) | 15.28 (14.59-16.17) | <.001 |
| INR, (0.8-1.25) | 1.08 (1.02-1.16) | 1.28 (1.10-1.47) | 1.07 (1.01-1.14) | <.001 |
| D-Dimer, mg/L (0-0.55) | 0.75 (0.38-1.71) | 3.52 (0.97-7.63) | 0.69 (0.36-1.49) | <.001 |
| PTA, (70-125) | 94.8 (90.3-98.5) | 82.8 (69.0-90.6) | 95.2 (91.1-98.8) | <.001 |
| **Myocardial injury markers, median (IQR)** | | | | |
| CK, IU/L (24-170) | 46.3 (32.6-72.4) | 95.0 (32.4-211.0) | 45.6 (32.5-67.7) | <.001 |
| CKMB, IU/L (0-24) | 9.0 (7.0-12.9) | 15.9 (10.2-26.5) | 8.7 (6.9-12.0) | <.001 |
| LDH, IU/L (120-250) | 202.0 (166.4-269.2) | 474.7 (344.5-703.3) | 198.2 (163.7-251.0) | <.001 |
| α-HBDH, IU/L (72-182) | 164.4 (134.6-227.5) | 371.0 (279.5-581.3) | 159.9 (133.2-208.0) | <.001 |

**Supplemental Table 8. Clinical characteristics and laboratory results of patients in severe group (continued)**

| **Variable** | **Total** | **Non-survivor** | **Survivor** | ***P* ^a^** |
| --- | --- | --- | --- | --- |
|  | **(n = 648)** | **(n = 64)** | **(n = 584)** |  |
| **Liver function indices, median (IQR)** | | | | |
| ALT, IU/L (7-40) | 21.9 (14.2-37.9) | 24.6 (14.7-44.1) | 21.7 (14.2-37.7) | 0.370 |
| AST, IU/L (7-45) | 20.80 (16.20-31.43) | 37.0 (22.8-53.1) | 20.2 (16.0-28.9) | <.001 |
| ALB, g/L (40-55) | 36.0 (31.8-39.0) | 31.2 (27.9-34.2) | 36.4 (32.2-39.2) | <.001 |
| GLB, g/L (20-30) | 27.3 (24.7-30.5) | 28.1 (24.9-31.3) | 27.2 (24.6-30.4) | 0.180 |
| TBIL, μmol/L (0-21) | 10.1 (7.7-13.5) | 14.1 (10.9-21.1) | 9.8 (7.5-12.8) | <.001 |
| DBIL, μmol/L (0-8) | 3.6 (2.7-5.4) | 6.8 (4.7-9.3) | 3.5 (2.6-4.9) | <.001 |
| IBIL, μmol/L (3.4-17) | 6.2 (4.7-8.1) | 7.2 (5.0-10.1) | 6.2 (4.7-8.0) | 0.044 |
| TBA, μmol/L (0-10) | 4 (2.6-6.3) | 4.7 (3.2-9.3) | 4.0 (2.5-6.2) | 0.078 |
| ALP, IU/L (35-135) | 73.4 (61.0-92.3) | 96.0 (70.8-115.4) | 72.8 (60.9-89.3) | <.001 |
| γ-GT, IU/L (7-45) | 33.4 (23.2-54.6) | 39.1 (24.6-73.9) | 33.2 (23.2-53.6) | 0.125 |
| **Kidney function indices, median (IQR)** | | | | |
| BUN, mmol/L (3.1-8.8) | 4.69 (3.79-6.20) | 8.63 (5.74-9.75) | 4.56 (3.72-5.92) | <.001 |
| Cr, μmol/L (41-81) | 65.4 (54.5-77.9) | 74.4 (61.7-110.2) | 64.6 (54.3-76.6) | <.001 |
| UA, μmol/L (142-340) | 269 (209-343) | 272 (190-395) | 268 (210-339) | 0.656 |
| CysC, mg/L (22-29) | 0.98 (0.86-1.15) | 1.34 (1.05-1.70) | 0.98 (0.86-1.12) | <.001 |
| **Infection-related indices, median (IQR)** | | | | |
| CRP, mg/L (0-4) | 4.83 (1.31-26.58) | 88.20 (44.14-145.44) | 3.81 (1.17-18.69) | <.001 |
| hsCRP, mg/L (0-4) | 5.31 (1.43-10.00) | 10.00 (10.00-17.14) | 4.30 (1.23-10.00) | <.001 |
| IL-6, pg/dL (< 0.07) | 0.039 (0.015-0.125) | 0.636 (0.304-1.487) | 0.030 (0.015-0.086) | <.001 |
| **Electrolytes and glucose, median (IQR)** | | | | |
| CO_2_, mmol/L (22-29) | 24.1 (22.6-25.8) | 22.1 (19.4-26.1) | 24.2 (22.8-25.7) | 0.002 |
| Na^+^, mmol/L (137-147) | 141.1 (139.1-143.1) | 140.9 (137.2-145.1) | 141.1 (139.2-143.1) | 0.956 |
| K^+^, mmol/L (3.5-5.3) | 4.21 (3.91-4.50) | 4.27 (3.80-4.69) | 4.20 (3.92-4.49) | 0.467 |
| Ca^2+^, mmol/L (211-252) | 213 (202-221) | 197 (190-209) | 214 (204-221) | <.001 |
| Cl^-^, mmol/L (99-110) | 105.8 (103.3-107.7) | 105.9 (100.1-110.1) | 105.7 (103.6-107.6) | 0.886 |
| Glu, mmol/L (3.9-6.1) | 5.25 (4.61-6.44) | 7.38 (5.68-9.75) | 5.13 (4.56-6.19) | <.001 |

Abbreviations: AF, atrial fibrillation; ALB, albumin; ALP, alkaline phosphatase; ALT, alanine transaminase; APTT, activated partial thromboplastin time; AST, aspartate transaminase; BASO, basophil; BUN, blood urea nitrogen; CHD, coronary heart disease; CK, creatine kinase; CKD, chronic kidney disease; CKMB, creative kinase MB; COPD, chronic obstructive pulmonary disease; Cr, creatinine; CRP, C-reactive protein; CVD, cerebrovascular disease; CysC, [cystatin](file:///D:\%E6%9C%89%E9%81%93%E8%AF%8D%E5%85%B8\Dict\7.5.2.0\resultui\dict\?keyword=cystatin)[C](file:///D:\%E6%9C%89%E9%81%93%E8%AF%8D%E5%85%B8\Dict\7.5.2.0\resultui\dict\?keyword=C); DBIL, direct bilirubin; EOS, eosinophil; FIB, fibrinogen; GLB, globulin; Glu, glucose; γ-GT, glutamyl transpeptidase; Hb, hemoglobin; α-HBDH, α-hydroxybutyrate dehydrogenase; hsCRP, high-sensitivity C-reactive protein; IBIL, indirect bilirubin; IL-6, interleukin-6; INR, international normalized ratio; LDH, lactate dehydrogenase; LYM, lymphocyte; MONO, monocyte; NEU, neutrophil; NLR, neutrophil-to-lymphocyte ratio; PLT, blood platelet; PT, prothrombin time; PTA, prothrombin activity; RBC, red blood cell; TBIL, [total](file:///D:\%E6%9C%89%E9%81%93%E8%AF%8D%E5%85%B8\Dict\7.5.2.0\resultui\dict\?keyword=total)[bilirubin](file:///D:\%E6%9C%89%E9%81%93%E8%AF%8D%E5%85%B8\Dict\7.5.2.0\resultui\dict\?keyword=bilirubin); TBA, total bile acid; TT, thrombin time; UA, [uric acid](javascript:;); WBC, white blood cell.

^a^ Compared between non-survivors and survivors in severe group.

**Supplemental Table 9. Univariate Cox regression and receiver operating characteristic analyses on the risk factors for mortality of patients in severe group**

| **Variable** | **Cox regression analysis** | | **ROC analysis** | | | |
| --- | --- | --- | --- | --- | --- | --- |
|  | **HR (95% CI)** | ***P* value** | **AUC (95% CI)** | ***P* value** | **Sensitivity** | **Specificity** |
| **Age, y** | 1.084 (1.058-1.110) | <.001 | 0.758 (0.702-0.813) | <.001 | 70.3% | 69.0% |
| **Male** | 1.179 (0.715-1.944) | 0.519 |  |  |  |  |
| **Comorbidity** | | | | | | |
| Hypertension | 1.469 (0.895-2.410) | 0.128 |  |  |  |  |
| Diabetes | 1.564 (0.896-2.730) | 0.116 |  |  |  |  |
| CHD | 3.487 (2.012-6.044) | <.001 | 0.591 (0.511-0.672) | 0.017 | 28.6% | 89.7% |
| AF | 4.041 (1.976-8.262) | <.001 | 0.559 (0.479-0.640) | 0.213 | 14.3% | 97.6% |
| CVD | 2.767 (1.443-5.306) | 0.002 | 0.558 (0.478-0.638) | 0.130 | 17.5% | 94.2% |
| COPD | 1.992 (0.941-4.215) | 0.072 |  |  |  |  |
| CKD | 7.680 (3.473-16.984) | <.001 | 0.550 (0.470-0.630) | 0.194 | 11.1% | 98.9% |
| Malignancy | 2.694 (1.226-5.922) | 0.014 | 0.536 (0.458-0.614) | 0.346 | 10.9% | 96.3% |
| **Blood routine test** | | | | | | |
| RBC, ×10^12^/L | 0.701 (0.456-1.077) | 0.105 |  |  |  |  |
| Hb, g/L | 0.986 (0.974-0.999) | 0.039 | 0.570 (0.483-0.657) | 0.073 | 39.3% | 81.3% |
| WBC, ×10^9^/L | 1.089 (1.056-1.123) | <.001 | 0.690 (0.601-0.779) | <.001 | 39.6% | 92.7% |
| NEU, ×10^9^/L | 1.100 (1.073-1.127) | <.001 | 0.779 (0.711-0.847) | <.001 | 79.4% | 70.0% |
| LYM, ×10^7^/L | 0.975 (0.968-0.982) | <.001 | 0.842 (0.788-0.896) | <.001 | 82.3% | 78.6% |
| MONO, ×10^7^/L | 0.964 (0.948-0.980) | <.001 | 0.708 (0.628-0.787) | <.001 | 60.7% | 73.8% |
| EOS, ×10^7^/L | 0.923 (0.885-0.962) | <.001 | 0.806 (0.740-0.872) | <.001 | 75.4% | 81.8% |
| BASO, ×10^7^/L | 0.656 (0.517-0.832) | 0.001 | 0.711 (0.636-0.786) | <.001 | 75.9% | 60.2% |
| NLR | 1.044 (1.035-1.054) | <.001 | 0.869 (0.815-0.922) | <.001 | 82.3% | 83.8% |
| PLT, ×10^9^/L | 0.983 (0.979-0.987) | <.001 | 0.806 (0.737-0.875) | <.001 | 72.4% | 77.7% |
| **Coagulation function test** | | | | | | |
| FIB, g/L | 0.539 (0.382-0.761) | <.001 | 0.575 (0.47-0.679) | 0.074 | 37.7% | 91.9% |
| APTT, s | 1.028 (1.020-1.036) | <.001 | 0.750 (0.668-0.833) | <.001 | 68.5% | 78.6% |
| PT, s | 1.052 (1.034-1.071) | <.001 | 0.768 (0.685-0.850) | <.001 | 58.5% | 90.2% |
| TT, s | 1.054 (1.039-1.069) | <.001 | 0.832 (0.767-0.896) | <.001 | 65.4% | 88.8% |
| INR | 1.796 (1.471-2.193) | <.001 | 0.792 (0.716-0.868) | <.001 | 56.6% | 93.6% |
| D-Dimer, mg/L | 1.105 (1.079-1.132) | <.001 | 0.779 (0.708-0.850) | <.001 | 61.5% | 83.6% |
| PTA | 0.943 (0.932-0.955) | <.001 | 0.838 (0.764-0.911) | <.001 | 68.2% | 89.8% |
| **Myocardial injury markers** | | | | | | |
| CK, IU/L | 1.003 (1.002-1.004) | <.001 | 0.709 (0.606-0.811) | <.001 | 69.6% | 78.7% |
| CKMB, IU/L | 1.008 (1.005-1.011) | <.001 | 0.779 (0.712-0.847) | <.001 | 60.4% | 85.6% |
| LDH, IU/L | 1.005 (1.004-1.005) | <.001 | 0.892 (0.828-0.955) | <.001 | 87.2% | 83.2% |
| α-HBDH, IU/L | 1.006 (1.004-1.007) | <.001 | 0.882 (0.819-0.946) | <.001 | 85.1% | 82.8% |

**Supplemental Table 9. Univariate Cox regression and receiver operating characteristic analyses on the risk factors for mortality of patients in severe group (continued)**

| **Variable** | **Cox regression analysis** | | **ROC analysis** | | | |
| --- | --- | --- | --- | --- | --- | --- |
|  | **HR (95% CI)** | ***P* value** | **AUC (95% CI)** | ***P* value** | **Sensitivity** | **Specificity** |
| **Liver function indices** | | | | | | |
| ALT, IU/L | 1.003 (0.999-1.007) | 0.118 |  |  |  |  |
| AST, IU/L | 1.007 (1.005-1.009) | <.001 | 0.723 (0.639-0.807) | <.001 | 69.2% | 73.3% |
| ALB, g/L | 0.863 (0.812-0.917) | <.001 | 0.760 (0.698-0.823) | <.001 | 75.4% | 67.1% |
| GLB, g/L | 1.037 (0.982-1.095) | 0.190 |  |  |  |  |
| TBIL, μmol/L | 1.048 (1.035-1.061) | <.001 | 0.713 (0.634-0.792) | <.001 | 67.9% | 69.4% |
| DBIL, μmol/L | 1.082 (1.059-1.106) | <.001 | 0.783 (0.710-0.855) | <.001 | 78.7% | 70.9% |
| IBIL, μmol/L | 1.103 (1.062-1.145) | <.001 | 0.588 (0.495-0.682) | 0.044 | 55.3% | 65.2% |
| TBA, μmol/L | 1.035 (1.005-1.066) | 0.022 | 0.578 (0.493-0.664) | 0.078 | 26.1% | 87.5% |
| ALP, IU/L | 1.011 (1.005-1.017) | <.001 | 0.658 (0.568-0.747) | <.001 | 57.8% | 74.6% |
| γ-GT, IU/L | 1.003 (0.999-1.008) | 0.169 |  |  |  |  |
| **Kidney function indices** | | | | | | |
| BUN, mmol/L | 1.191 (1.146-1.238) | <.001 | 0.798 (0.723-0.873) | <.001 | 80.8% | 71.5% |
| Cr, μmol/L | 1.009 (1.007-1.012) | <.001 | 0.659 (0.577-0.742) | <.001 | 34.0% | 91.8% |
| UA, μmol/L | 1.004 (1.002-1.006) | <.001 | 0.520 (0.419-0.621) | 0.656 | 23.4% | 91.8% |
| CysC, mg/L | 2.865 (2.053-3.998) | <.001 | 0.737 (0.650-0.824) | <.001 | 78.0% | 62.6% |
| **Infection-related indices** | | | | | | |
| CRP, mg/L | 1.016 (1.012-1.019) | <.001 | 0.888 (0.846-0.930) | <.001 | 79.5% | 84.0% |
| hsCRP, mg/L | 1.012 (1.008-1.016) | <.001 | 0.822 (0.777-0.867) | <.001 | 89.3% | 65.9% |
| IL-6, pg/dL | 1.114 (1.062-1.168) | <.001 | 0.914 (0.876-0.952) | <.001 | 87.5% | 84.7% |
| **Electrolytes and glucose** | | | | | | |
| CO_2_, mmol/L | 0.848 (0.788-0.913) | <.001 | 0.636 (0.531-0.741) | 0.002 | 51.1% | 82.4% |
| Na^+^, mmol/L | 1.075 (1.021-1.132) | 0.006 | 0.502 (0.401-0.603) | 0.956 | 27.8% | 91.2% |
| K^+^, mmol/L | 1.411 (0.909-2.189) | 0.125 |  |  |  |  |
| Ca^2+^, mmol/L | 0.950 (0.927-0.974) | <.001 | 0.772 (0.690-0.853) | <.001 | 74.5% | 72.7% |
| Cl^-^, mmol/L | 1.061 (1.003-1.122) | 0.040 | 0.506 (0.391-0.622) | 0.886 | 38.3% | 85.9% |
| Glu, mmol/L | 1.185 (1.118-1.255) | <.001 | 0.768 (0.703-0.832) | <.001 | 85.2% | 60.6% |

Abbreviations: AF, atrial fibrillation; ALB, albumin; ALP, alkaline phosphatase; ALT, alanine transaminase; APTT, activated partial thromboplastin time; AST, aspartate transaminase; AUC, area under the curve; BASO, basophil; BUN, blood urea nitrogen; CHD, coronary heart disease; CK, creatine kinase; CKD, chronic kidney disease; CKMB, creative kinase MB; COPD, chronic obstructive pulmonary disease; Cr, creatinine; CRP, C-reactive protein; CVD, cerebrovascular disease; CysC, [cystatin](file:///D:\%E6%9C%89%E9%81%93%E8%AF%8D%E5%85%B8\Dict\7.5.2.0\resultui\dict\?keyword=cystatin)[C](file:///D:\%E6%9C%89%E9%81%93%E8%AF%8D%E5%85%B8\Dict\7.5.2.0\resultui\dict\?keyword=C); DBIL, direct bilirubin; EOS, eosinophil; FIB, fibrinogen; GLB, globulin; Glu, glucose; γ-GT, glutamyl transpeptidase; Hb, hemoglobin; α-HBDH, α-hydroxybutyrate dehydrogenase; HR, hazard ratio; hsCRP, high-sensitivity C-reactive protein; IBIL, indirect bilirubin; IL-6, interleukin-6; INR, international normalized ratio; LDH, lactate dehydrogenase; LYM, lymphocyte; MONO, monocyte; NEU, neutrophil; NLR, neutrophil-to-lymphocyte ratio; PLT, blood platelet; PT, prothrombin time; PTA, prothrombin activity; RBC, red blood cell; ROC, receiver operating characteristic curve; TBIL, [total](file:///D:\%E6%9C%89%E9%81%93%E8%AF%8D%E5%85%B8\Dict\7.5.2.0\resultui\dict\?keyword=total)[bilirubin](file:///D:\%E6%9C%89%E9%81%93%E8%AF%8D%E5%85%B8\Dict\7.5.2.0\resultui\dict\?keyword=bilirubin); TBA, total bile acid; TT, thrombin time; UA, [uric acid](javascript:;); WBC, white blood cell.

**Supplemental Table 10. Multivariate Cox regression analysis on the risk factors for mortality of patients in severe group**

| **Variable** | **HR (95% CI)** | ***P* value** |
| --- | --- | --- |
| CKD | 9.638 (1.414-65.702) | 0.021 |
| NLR | 1.060 (1.020-1.101) | 0.001 |
| DBIL, μmol/L | 1.147 (1.065-1.236) | <.001 |
| IL-6, pg/dL | 1.151 (1.059-1.250) | 0.001 |

Abbreviations: AF, atrial fibrillation; AUC, area under the curve; CHD, coronary heart disease; CKD, chronic kidney disease; CVD, cerebrovascular disease; CysC, [cystatin](file:///D:\%E6%9C%89%E9%81%93%E8%AF%8D%E5%85%B8\Dict\7.5.2.0\resultui\dict\?keyword=cystatin)[C](file:///D:\%E6%9C%89%E9%81%93%E8%AF%8D%E5%85%B8\Dict\7.5.2.0\resultui\dict\?keyword=C); DBIL, direct bilirubin; HR, hazard ratio; IL-6, interleukin-6; LDH, lactate dehydrogenase; NLR, neutrophil-to-lymphocyte ratio; PTA,[prothrombin activity](javascript:;).

The model contains 12 variables, including all significant demographic variables (age, CHD, AF, CVD, CKD, and malignancy) identified in the univariate analysis, as well as one variable with high AUC in each category of laboratory tests (NLR, PTA, LDH, DBIL, CysC, and IL-6).

**Supplemental Table 11. Comparison of possible prognostic models for mortality of patients in severe grou**p

| **Models ^a^** | **15-d AUC (95% CI)** | **30-d AUC (95% CI)** | **60-d AUC (95% CI)** | **LR χ2** | **df** | **C-index (95% CI)** |
| --- | --- | --- | --- | --- | --- | --- |
| Model 1: LP = CKD | 0.540 (0.444-0.635) ^b^ | 0.556 (0.472-0.640) ^b^ | 0.550 (0.470-0.630) ^b^ | 15.79 | 1 | 0.545 (0.543-0.547) |
| Model 2: LP = NLR | 0.859 (0.791-0.927) | 0.861 (0.802-0.919) | 0.869 (0.815-0.922) | 43.20 | 1 | 0.832 (0.805-0.859) |
| Model 3: LP = DBIL | 0.769 (0.673-0.865) | 0.776 (0.694-0.857) | 0.783 (0.710-0.855) | 27.17 | 1 | 0.759 (0.717-0.801) |
| Model 4: LP = IL-6 | 0.908 (0.865-0.952) | 0.911 (0.869-0.954) | 0.914 (0.876-0.952) | 9.55 | 1 | 0.894 (0.872-0.916) |
| Model 5: LP = 0.140 × IL-6 + 2.266 × CKD | 0.907 (0.863-0.950) | 0.915 (0.872-0.959) | 0.917 (0.878-0.955) | 23.41 | 2 | 0.896 (0.875-0.917) |
| Model 6: LP = 0.140 × IL-6 + 0.058 × NLR | 0.924 (0.884-0.965) | 0.925 (0.890-0.961) | 0.926 (0.893-0.959) | 38.41 | 2 | 0.891 (0.869-0.913) |
| Model 7: LP = 0.140 × IL-6 + 0.137 × DBIL | 0.780 (0.637-0.924) ^c^ | 0.776 (0.654-0.899) | 0.795 (0.700-0.891) | 20.77 | 2 | 0.758 (0.695-0.821) |
| Model 8: LP = 0.140 × IL-6 + 2.266 × CKD + 0.137 × DBIL + 0.058 × NLR | 0.897 (0.797-0.998) | 0.908 (0.831-0.986) | 0.912 (0.852-0.972) | 48.49 | 4 | 0.884 (0.839-0.929) |

Abbreviations: AUC, area under the curve; CKD, chronic kidney disease; DBIL, direct bilirubin; df, degree of freedom; HL χ2: Hosmer-Lemeshow χ2; IL-6, interleukin-6; LP: linear predictor; LR χ2: likelyhood ratio χ2; NLR, neutrophil-to-lymphocyte ratio.

^a^ The coefficients of variables in Models 5-8 were derived from multivariate Cox regression analysis.

**^b^** *P* value > 0.05.

**^c^** *P* value < 0.01.

If not specifically denoted, *P* values of AUCs or LR χ2 in the models < 0.001.

**Supplemental Table 12. Comparison of the performance between current and previous models for mortality of COVID-19**

| **Model** | **Sample size ^a^** | **Variables** | **C-index (95% CI)** | **AUC (95% CI)** | **Sensitivity** | **Specificity** |
| --- | --- | --- | --- | --- | --- | --- |
| ([5](#_ENREF_5)) | 51/110 | PT, BUN, WBC, IL-2R, DBIL, MYO, FDP | − | 0.997 (0.990-1.000) | 93% | 91% |
| ([6](#_ENREF_6)) | 21/120 | CRP, cTnl, PaO_2_/FiO_2_ | − | 0.988 (0.972-1.000) | − | − |
| ([7](#_ENREF_7)) | 54/108 | NEU, LYM, PLT, IL-2R | − | 0.964 (0.909-0.990) | 90.74% | 94.44% |
| ([8](#_ENREF_8)) | 51/739 | IL-8, CD4^+^ T cell, NK cell | − | 0.956 | 90.20% | 90.26% |
| ([9](#_ENREF_9)) | 129/1115 | PAB, NEU, LYM, PCT, hsCRP, PT, LDH, Cr, cTnI | − | 0.955 (0.941–0.970) | 86.82% | 90.37% |
| Current study | 100/3342 | IL-6 | 0.936 (0.925-0.947) | 0.953 (0.934-0.972) | 91.8% | 86.3% |
| ([10](#_ENREF_10)) | 78/450 | WBC, PNI, LDH |  | 0.950 (0.922-0.978) |  |  |
| ([11](#_ENREF_11)) | 85/305 | BUN, D-dimer | 0.940 | 0.940 (0.900-0.970) | 85% | 91% |
| ([12](#_ENREF_12)) | 36/501 | Age, PaO_2_/FiO_2_, NLR, LDH, IL-6 | − | 0.940 (0.890-1.000) | 88% | 89% |
| ([13](#_ENREF_13)) | 14/244 | Age, sex, hypertension, diabetes, GFR, cTnI | − | 0.925 (0.873-0.978) | − | − |
| ([14](#_ENREF_14)) | 50/1590 | Age, CHD, CVD, dyspnea, PCT, AST | 0.910 (0.850-0.970) | − | − | − |
| ([15](#_ENREF_15)) | 69/390 | Hypertension, NLR, NT-proBNP | 0.901 | 0.922 | − | − |
| ([16](#_ENREF_16)) | 21/176 | Age, NLR, D-dimer, CRP | − | 0.921 (0.835-0.968) | -− | − |
| ([17](#_ENREF_17)) | 201/1018 | IL-6, CD8^+^ T cell | − | 0.907 (0.886-0.928) |  |  |
| ([18](#_ENREF_18)) | 96/516 | Age, LDH, NLR, DBIL | 0.886 (0.873-0.899) | − | − | − |
| ([19](#_ENREF_19)) | 62/297 | Age, HF, PCT, LDH, COPD, SpO_2_, HR | − | 0.830 (0.830-0.920) | 7.1% | 100% |
| ([20](#_ENREF_20)) | 140/707 | Age, Myocardial injury, Cr, CRP, CCI | 0.775 | 0.790 | − | − |
| ([21](#_ENREF_21)) | 212/1663 | Age, sex, diabetes, LYM, PCT | − | 0.765 (0.725-0.805) | 82.1% | 53% |
| ([22](#_ENREF_22)) **^b^** | 49/113 | Age, CHD, LYM, PCT, D-dimer | − | 0.919 (0.870-0.970) | − | − |
| Current study **^b^** | 64/648 | IL-6 | 0.894 (0.872-0.916) | 0.914 (0.876-0.952) | 87.5% | 84.7% |
| ([23](#_ENREF_23)) **^b^** | 130/256 | Age, hsCRP, LYM, D-dimer |  | 0.881 | 83.9% | 79.4% |
| ([24](#_ENREF_24)) **^c^** | 34/336 | LYM, BUN, D-dimer |  | 0.994 (0.979-0.999) | 100% | 97.2% |
| ([25](#_ENREF_25)) **^c^** | 50/104 | Age, chest tightness, AST, BUN | − | 0.893 (0.807-0.980) | 96% | 74.1% |

Abbreviations: AST, aspartate transaminase; BUN, blood urea nitrogen; CHD, coronary heart disease; CRP, C reactive protein; CVD, cerebrovascular disease; CCl, Charlson comorbidity index; COPD, chronic obstructive pulmonary disease; Cr, creatinine; CRP, C-reactive protein; cTnI, cardiac troponin I; DBIL, direct bilirubin; FDP, fibrinogen degradation product; GFR, glomerular filtration rate; HF, heart failure; HR heart rate; hsCRP, high-sensitivity C-reactive protein; IL-2R, interleukin-2 receptor, IL-6, interleukin-6; IL-8 interleukin-8; LDH, lactate dehydrogenase; LYM, lymphocytes; MYO, myoglobin; NEU, neutrophils; NLR, neutrophil-to-lymphocyte ratio; NT-proBNP, N-terminal pro-B-type natriuretic peptide; PAB, pre-albumin; PaO_2_/FiO_2_, partial pressure of O_2_/fraction of inspiration O_2_; PCT, pro-calcitonin; PNI, prognostic nutritional index; PLT, platelets; PT, prothrombin time; WBC, white blood cell.

^a^ The number of deceased patients/ the number of all patients in the cohort.

^b^ Mortality of patients in severe group.

^c^ Mortality of patients of critical subtype.

**References:**

1. Wada H, Thachil J, Di Nisio M, Mathew P, Kurosawa S, Gando S, et al. Guidance for diagnosis and treatment of DIC from harmonization of the recommendations from three guidelines. Journal of thrombosis and haemostasis : JTH. 2013.

2. Thygesen K, Alpert JS, Jaffe AS, Chaitman BR, Bax JJ, Morrow DA, et al. Fourth Universal Definition of Myocardial Infarction (2018). Circulation. 2018;138(20):e618-e51.

3. Chen G, Wu D, Guo W, Cao Y, Huang D, Wang H, et al. Clinical and immunological features of severe and moderate coronavirus disease 2019. The Journal of clinical investigation. 2020;130(5):2620-9.

4. Kidney Disease: Improving Global Outcomes (KDIGO) Acute Kidney Injury Work Group. KDIGO clinical practice guideline for Acute Kidney Injury. idney Int Suppl.2012;2:1–138.

5. Wu G, Zhou S, Wang Y, Lv W, Wang S, Wang T, et al. A prediction model of outcome of SARS-CoV-2 pneumonia based on laboratory findings. Scientific reports. 2020;10(1):14042.

6. Pan D, Cheng D, Cao Y, Hu C, Zou F, Yu W, et al. A Predicting Nomogram for Mortality in Patients With COVID-19. Frontiers in public health. 2020;8:461.

7. Wang F, Hou H, Wang T, Luo Y, Tang G, Wu S, et al. Establishing a model for predicting the outcome of COVID-19 based on combination of laboratory tests. Travel medicine and infectious disease. 2020;36:101782.

8. Luo Y, Mao L, Yuan X, Xue Y, Lin Q, Tang G, et al. Prediction Model Based on the Combination of Cytokines and Lymphocyte Subsets for Prognosis of SARS-CoV-2 Infection. Journal of clinical immunology. 2020:1-10.

9. Luo Y, Xue Y, Mao L, Yuan X, Lin Q, Tang G, et al. Prealbumin as a Predictor of Prognosis in Patients With Coronavirus Disease 2019. Frontiers in medicine. 2020;7:374.

10. Wang R, He M, Yin W, Liao X, Wang B, Jin X, et al. The Prognostic Nutritional Index is associated with mortality of COVID-19 patients in Wuhan, China. Journal of clinical laboratory analysis. 2020:e23566.

11. Cheng A, Hu L, Wang Y, Huang L, Zhao L, Zhang C, et al. Diagnostic performance of initial blood urea nitrogen combined with D-dimer levels for predicting in-hospital mortality in COVID-19 patients. International journal of antimicrobial agents. 2020;56(3):106110.

12. Laguna-Goya R, Utrero-Rico A, Talayero P, Lasa-Lazaro M, Ramirez-Fernandez A, Naranjo L, et al. IL-6-based mortality risk model for hospitalized patients with COVID-19. The Journal of allergy and clinical immunology. 2020.

13. Cao J, Zheng Y, Luo Z, Mei Z, Yao Y, Liu Z, et al. Myocardial injury and COVID-19: Serum hs-cTnI level in risk stratification and the prediction of 30-day fatality in COVID-19 patients with no prior cardiovascular disease. Theranostics. 2020;10(21):9663-73.

14. Chen R, Liang W, Jiang M, Guan W, Zhan C, Wang T, et al. Risk Factors of Fatal Outcome in Hospitalized Subjects With Coronavirus Disease 2019 From a Nationwide Analysis in China. Chest. 2020;158(1):97-105.

15. Dong YM, Sun J, Li YX, Chen Q, Liu QQ, Sun Z, et al. Development and Validation of a Nomogram for Assessing Survival in Patients with COVID-19 Pneumonia. Clinical infectious diseases : an official publication of the Infectious Diseases Society of America. 2020.

16. Weng Z, Chen Q, Li S, Li H, Zhang Q, Lu S, et al. ANDC: an early warning score to predict mortality risk for patients with Coronavirus Disease 2019. Journal of translational medicine. 2020;18(1):328.

17. Luo M, Liu J, Jiang W, Yue S, Liu H, Wei S. IL-6 and CD8+ T cell counts combined are an early predictor of in-hospital mortality of patients with COVID-19. JCI insight. 2020;5(13).

18. Zhang S, Guo M, Duan L, Wu F, Hu G, Wang Z, et al. Development and validation of a risk factor-based system to predict short-term survival in adult hospitalized patients with COVID-19: a multicenter, retrospective, cohort study. Critical care (London, England). 2020;24(1):438.

19. Zhao Z, Chen A, Hou W, Graham JM, Li H, Richman PS, et al. Prediction model and risk scores of ICU admission and mortality in COVID-19. PloS one. 2020;15(7):e0236618.

20. Lorente-Ros A, Monteagudo Ruiz JM, Rincón LM, Ortega Pérez R, Rivas S, Martínez-Moya R, et al. Myocardial injury determination improves risk stratification and predicts mortality in COVID-19 patients. Cardiology journal. 2020.

21. Yu C, Lei Q, Li W, Wang X, Liu W, Fan X, et al. Clinical Characteristics, Associated Factors, and Predicting COVID-19 Mortality Risk: A Retrospective Study in Wuhan, China. American journal of preventive medicine. 2020;59(2):168-75.

22. Shang Y, Liu T, Wei Y, Li J, Shao L, Liu M, et al. Scoring systems for predicting mortality for severe patients with COVID-19. EClinicalMedicine. 2020;24:100426.

23. Hu C, Liu Z, Jiang Y, Shi O, Zhang X, Xu K, et al. Early prediction of mortality risk among patients with severe COVID-19, using machine learning. International journal of epidemiology. 2020.

24. Liu Q, Song NC, Zheng ZK, Li JS, Li SK. Laboratory findings and a combined multifactorial approach to predict death in critically ill patients with COVID-19: a retrospective study. Epidemiology and infection. 2020;148:e129.

25. Wang B, Zhong F, Zhang H, An W, Liao M, Cao Y. Risk factors analysis and nomogram construction of non-survivors in critical patients with COVID-19. Japanese journal of infectious diseases. 2020.
